# Utilization of CRISPR-Cas genome editing technology in filamentous fungi: function and advancement potentiality

**DOI:** 10.3389/fmicb.2024.1375120

**Published:** 2024-03-28

**Authors:** Qiqing Shen, Haihua Ruan, Hongyang Zhang, Tao Wu, Kexin Zhu, Wenying Han, Rui Dong, Tianwei Ming, Haikun Qi, Yan Zhang

**Affiliations:** Tianjin Key Laboratory of Food Science and Biotechnology, College of Biotechnology and Food Science, Tianjin University of Commerce, Tianjin, China

**Keywords:** filamentous fungi, CRISPR-Cas, gene editing, CRISPR-dCas, base editors, Prime editors

## Abstract

Filamentous fungi play a crucial role in environmental pollution control, protein secretion, and the production of active secondary metabolites. The evolution of gene editing technology has significantly improved the study of filamentous fungi, which in the past was laborious and time-consuming. But recently, CRISPR-Cas systems, which utilize small guide RNA (sgRNA) to mediate clustered regularly interspaced short palindromic repeats (CRISPR) and CRISPR-associated proteins (Cas), have demonstrated considerable promise in research and application for filamentous fungi. The principle, function, and classification of CRISPR-Cas, along with its application strategies and research progress in filamentous fungi, will all be covered in the review. Additionally, we will go over general matters to take into account when editing a genome with the CRISPR-Cas system, including the creation of vectors, different transformation methodologies, multiple editing approaches, CRISPR-mediated transcriptional activation (CRISPRa) or interference (CRISPRi), base editors (BEs), and Prime editors (PEs).

## 1 Introduction

Most filamentous fungi belong to the phyla Ascomycetes and Basidiomycetes. These microorganisms are of great significance to humans and are widely distributed in nature. Some specific species, including *Aspergillus fumigatus, Trichoderma asperellum, Aspergillus niger, Penicillium simplicissimum, Penicillium janthinellum*, and *Penicillium simplicissimum*, have been found to have a high capacity for adsorbing heavy metals and can play a key role in controlling environmental pollution (Dusengemungu et al., 2020). Additionally, other species like *Beauveria* and *Metarhizium* have been utilized as biological pesticides to control pests (Sullivan et al., 2022). Furthermore, the production of various enzymes and organic acids, which have multiple roles in industry, medicine, and food, has been attributed to filamentous fungi, including *Aspergillus* and *Trichoderma* (Wosten, 2019). These fungi have also been employed over a century as adaptable and extremely productive cell factories, synthesizing a variety of secondary metabolites with significant biological properties. For example, penicillin produced by *Penicillium* (Guzmán-Chávez et al., 2018), cephalosporin generated by *Penicillium chrysogenum* (Liu L. et al., 2022), lovastatin generated by *Aspergillus* (Subhan et al., 2016), cordycepin generated by *Cordyceps* (Ashraf et al., 2020), etc. (Table 1).

**Table 1:** Examples of filamentous fungi employed in the synthesis of enzymes and small-molecule compounds.

Products		Applications	Host
Enzymes	Amylase	Bread making and production of glucose syrup	*Aspergillus niger*, *Aspergillus oryzae*
	Protease	Food, laundry detergent, leather and pharmaceuticals pectinase	*Aspergillus niger*, *Aspergillus clavatus*
	Pectinase	Clearing of juices and wine	*Aspergillus niger, Aspergillus oryzae*
	Cellulase	Fabric softener	*Trichoderma viride*
Organic acids	Citric acid	Food, beverage, cosmetics, laundry detergent	*Aspergillus niger*
	Itaconic acid	Medicine, chemical industry	*Aspergillus terreus*
	Malic acid	Food, medicine, cosmetics	*Ustilago trichophora*, *Aspergillus oryzae*
	Lysergic acid	Treatment of dementia, dementia and hyperprolactinaemia	*Metarhizium brunneum*
	Gibberellic acids (GA)	Broad-spectrum plant growth regulators	*Fusarium fujikuroi*
	Galactaric acid from D-galacturonic acid	Skincare products	*Aspergillus niger*
	Gluconic acid	Food, medicine	*Aspergillus niger*
Secondary metabolites	Penicillin	Antibiotics, mainly against gram-positive bacteria	*Penicillium chrysogenum*
	Monascus Red pigment	Natural edible pigment	*Monascus purpureus*
	Cephalosporin	Broad-spectrum antibiotics	*Acremonium chrysogenum*
	Lovastatin	Treatment of hypercholesterolemia	*Aspergillus terreus*
	Pentostatin	Treatment of tumors	*Streptomyces antiboticus*
	Cordycepin	Adenosine antibiotics, treatment of tumors and diabetes	*Cordyceps militaris*
	Echinocandin	Antifungal drugs	*Aspergillus nidulans*
	Beauvericin	Pesticides	*beauveria bassiana*

Up to now, three main gene editing technologies: zinc finger nucleases (ZFNs) (Kim et al., 1996), transcription activator-like effector nucleases (TALENs) (Miller et al., 2011), and the CRISPR-Cas system, which is constructed from clustered regularly spaced short palindromic repeats (CRISPR) and CRISPR-associated proteins (Cas) (Jinek et al., 2012) have been widely used, among which the CRISPR-Cas system, considered the third-generation genome editing tool, earns a dominant spot in the field of gene editing due to its numerous benefits, including simpler design, lower cost, higher targeting efficiency, lower off-target rate, and lower cytotoxicity (Gupta et al., 2019).

**Figure 1:**
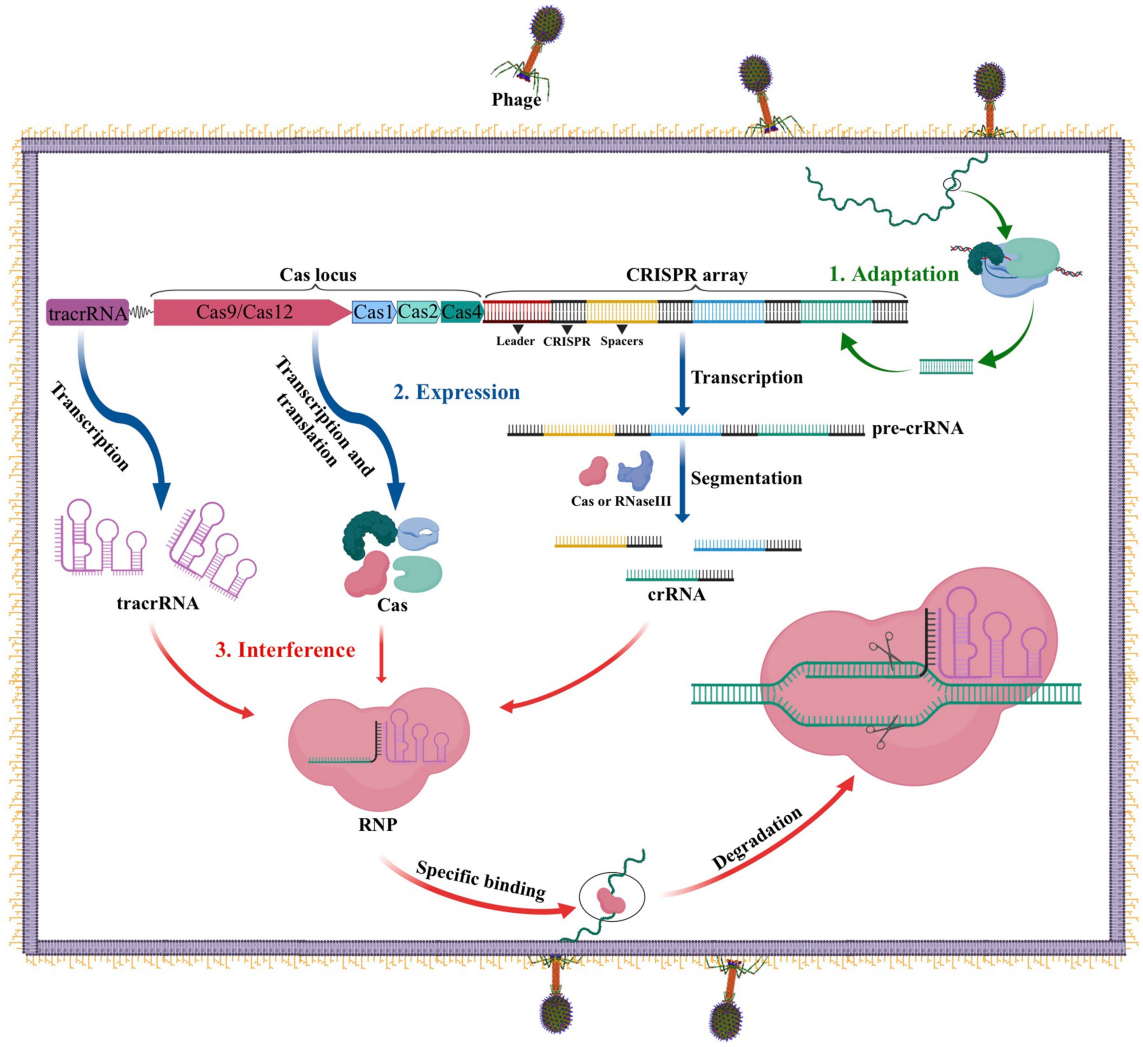
The CRISPR-Cas adaptive immune system in prokaryotes. 1. Acquisition: In this stage, the invading DNA is fragmented and a new protospacer is selected and integrated into the CRISPR array as a new spacer. 2. Expression: During this second stage, the pre-crRNA produced by the CRISPR array is cleaved into mature crRNAs by RNase III. These mature crRNAs, along with tracrRNAs and Cas proteins, assemble to form ribonucleoprotein (RNP) complexes. 3. Interference: In the final stage, the crRNP uses a small guide RNA (sgRNA) (composed of crRNA and tracrRNA) to identify invading DNA, and Cas proteins cut the foreign DNA, removing the foreign genetic material.

The CRISPR-Cas system performs an important function as the adaptive immune system in prokaryotes such as bacteria and archaea to fend off infections from viruses, phages, and other foreign substances. Three stages comprise this immune defense: adaptation, expression, and interference (Carter and Wiedenheft, 2015). As illustrated in Figure 1, during the adaptation stage, specific Cas proteins identify and cut the DNA of the invading virus or phage into short fragments, which are subsequently added to the CRISPR locus array as spacers between two repeats. In the expression stage, if the same DNA invades the bacteria again, the leader sequence located at the CRISPR locus initiates CRISPR transcription, resulting in the formation of a precursor CRISPR-derived RNA (pre-crRNA). This pre-crRNA is then cleaved by either ribonuclease (RNase III) or a Cas protein at the resequencing site, producing mature CRISPR RNA (crRNA). Finally, in the interference process, the mature crRNA combines with Cas to build a ribonucleoprotein (RNP), which can identify and cleave foreign DNA that complements the crRNA. This process ultimately leads to the degradation of viral nucleic acid and the successful defense against infection (Carter and Wiedenheft, 2015).

Double-strand breaks (DSBs) caused by the CRISPR-Cas system can activate either non-homologous end joining (NHEJ) or homology-directed repair (HDR) in the host (Doudna and Charpentier, 2014). NHEJ is the primary repair pathway in the absence of exogenous DNA fragments, resulting in random base substitution, insertion, and loss at the breakage points. However, the HDR pathway utilizes exogenous donor fragments to precisely edit the target gene (Ma et al., 2016). In this paper, several important CRISPR-Cas systems and the latest advancements in CRISPR-Cas-mediated genome editing systems in filamentous fungi are briefly reviewed.

## 2 CRISPR-Cas systems

**Figure 2:**
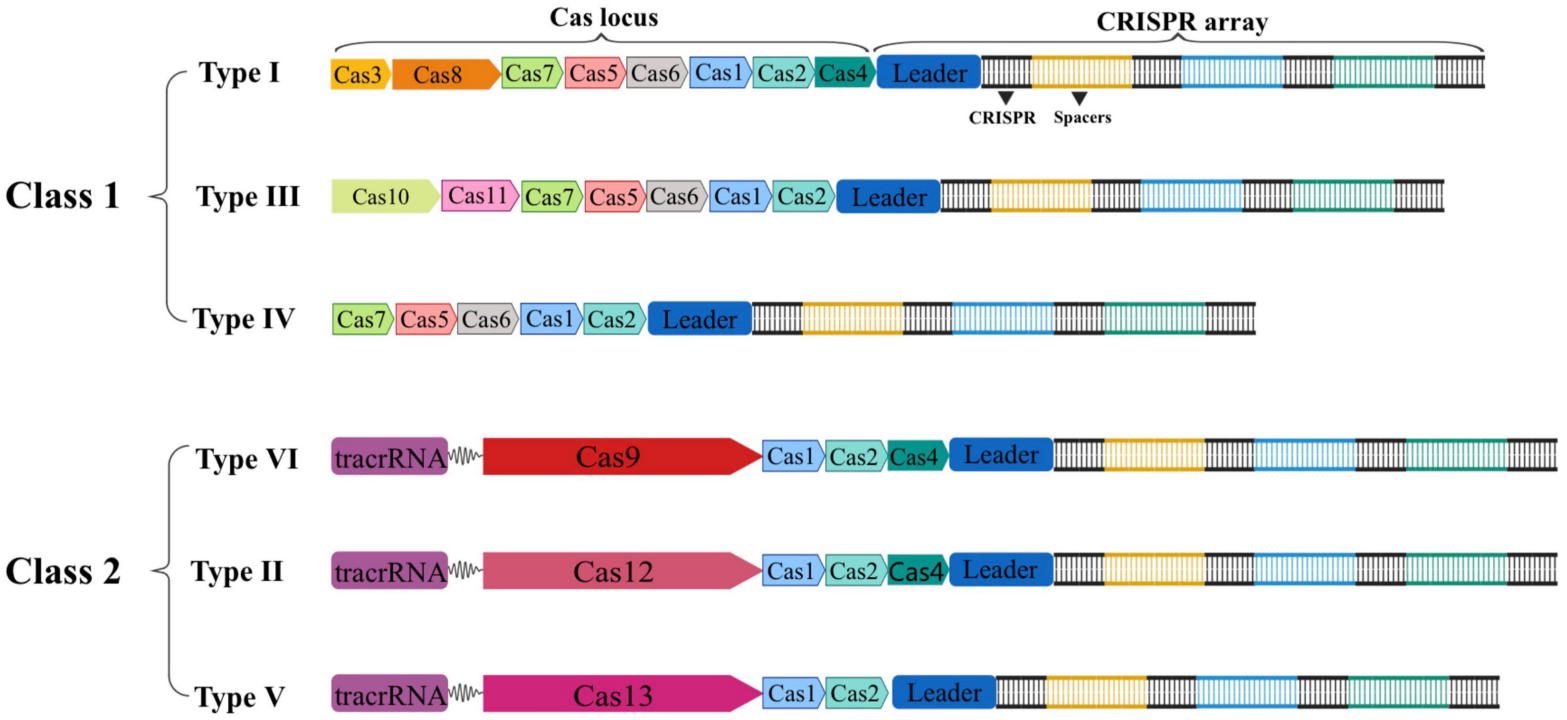
Schematic of the two classes of CRISPR-Cas systems general composition of locus. Class 1 CRISPR-Cas systems are composed of multiple Cas proteins that form an effector complex responsible for binding and processing the target crRNA. In contrast, class 2 systems have a single, multidomain crRNA-binding protein that performs the same function as the entire effector complex in class 1.

The CRISPR-Cas gene editing technology has developed rapidly throughout the past two decades. In 2011, the National Center for Biotechnology Information (NCBI) proposed a new classification method with Cas1 and Cas2 genes serving as the system’s fundamental components. Since then, Makarova et al. have classified or updated the CRISPR-Cas system three times in 2011, 2015, and 2020, respectively (Makarova et al., 2011, 2015, 2020). In the latest classification, the CRISPR-Cas system is separated into two major classes based on the composition of Cas proteins and the properties of effector complexes. It is then further divided into 6 types and 33 subtypes based on the sequence and functional characteristics of splicing modules (Makarova et al., 2020). As shown in Figure 2, the effector complexes of the Class 1 system are made up of 4–8 Cas protein subunits. The common feature of the Class 1 system is that multiple Cas protein effector complexes are used to interfere with target nucleic acids, including three types and 16 subtypes: type I, type III, and type IV. On the other hand, the effector complexes of the Class 2 system are single multidomain proteins, such as Cas9, Cas12, and Cas13, and are separated into three types: type II, type V, and type VI, with 17 subtypes in total (Makarova et al., 2020). Due to the advantages of single nucleases, the second type of CRISPR-Cas system is more widely used in the eukaryotic gene editing field than the first type of system. Therefore, we will focus on Cas9, Cas12, and Cas13.

### 2.1 CRISPR-Cas9 system

**Figure 3:**
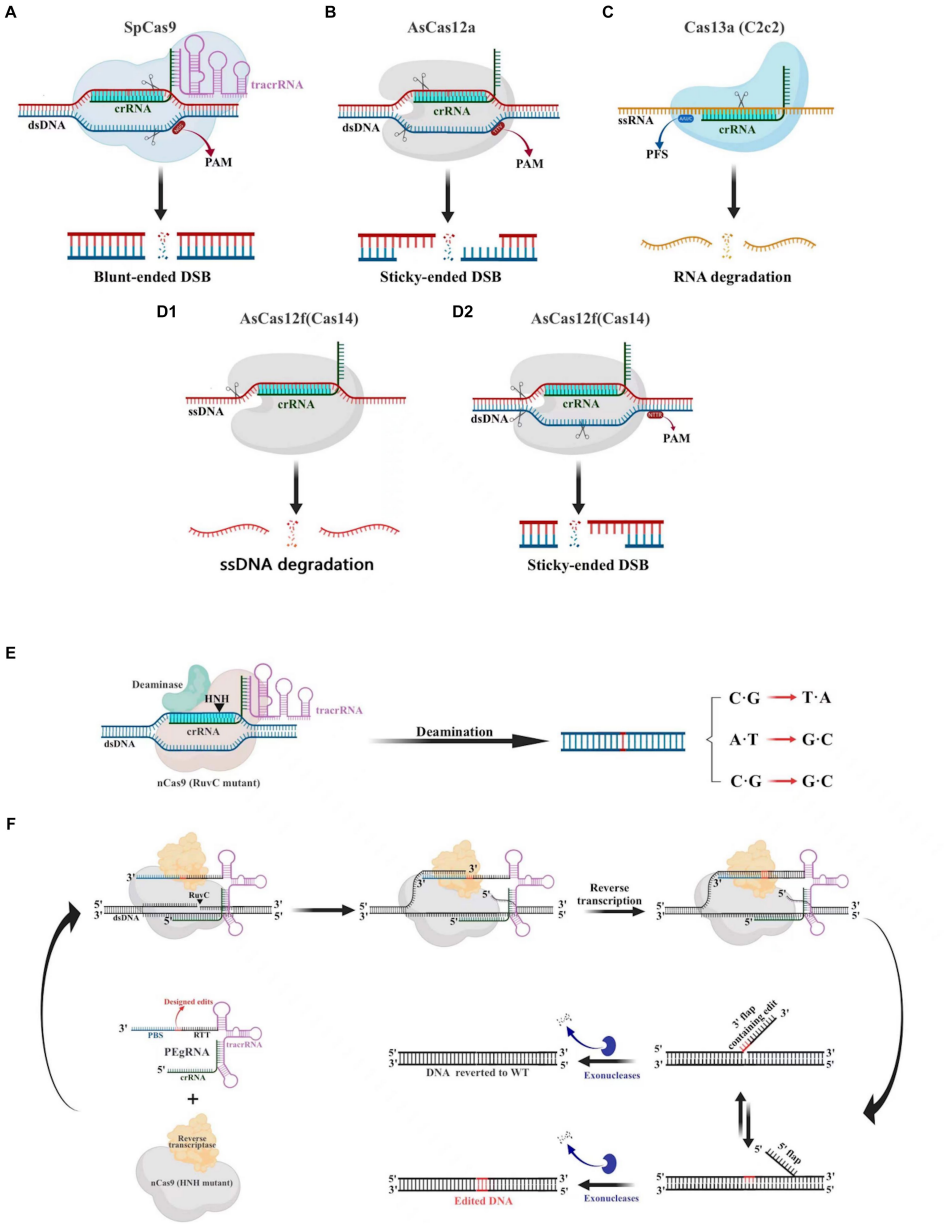
Gene editing schematic of four commonly used CRISPR-Cas systems (Cas9, Cas12a Cas13a and AsCas12f). **(A)** The CRISPR-Cas9 system is capable of cleaving double-stranded DNA (dsDNA) 3 bp upstream of the protospacer adjacent motif (PAM) under the guidance of a single guide RNA (sgRNA), resulting in a blunt double-stranded breaks (DSBs) at the target site. **(B)** The CRISPR-Cas12a system relies on crRNA to recognize the PAM of dsDNA, which then leads to the cleavage of the target DNA and the production of sticky-ended DSBs. **(C)** The CRISPR-Cas13a system utilizes crRNA to specifically target and cleave single-stranded RNA downstream of the PFS. **(D)** The CRISPR/AsCas12f system has the unique ability to cleave single-stranded DNA (ssDNA) without the need for PAM. Additionally, it is capable of cleaving dsDNA to create sticky-ended DSBs, which is achieved through the recognition of PAM sites. **(E)** Base editors can generate C·G to T·A, A·T to G·C, C·G to G·C point mutations without creating DSB. **(F)** Schematic diagram illustrating the components involved in the PE mechanism.

The CRISPR-Cas9 system consists of the Cas9 nuclease and small guide RNA (sgRNA). Numerous cells, animals, and plants have been edited using this potent and promising gene editing system (Huang et al., 2022). The CRISPR-Cas9 system (Figure 3A), which is derived from *Streptococcus pyogenes* (SpCas9), is one of the most characterized, most commonly utilized, and most active Cas enzymes among the CRISPR-Cas systems (Hsu et al., 2013). SpCas9 is a large protein (1,368 amino acids) with two distinct lobes in its apo state: the alpha-helical recognition (REC) lobe, composed of three alpha-helical domains (Hel-I, Hel-II, and Hel-III), while the nuclease (NUC) lobe retains the conserved HNH, the split RuvC nuclease domains, and the more variable C-terminal domain (CTD). The elongated CTD also displays a Cas9-specific fold and contains PAM-interacting sites required for protospacer-adjacent motifs (PAM) interrogation (Jiang et al., 2017). The sgRNA is designed by fusing crRNA with trans-activating crRNA (tracrRNA). It is an RNA complex containing a human-designed 20-bp nucleotide sequence complementary to the target gene at the 5′ end, followed by a repeat of crRNA and an inverted repeat of tracrRNA (Prasad et al., 2021). During gene editing, a ribonucleoprotein composed of the Cas9 protein and sgRNA recognizes the PAM and target sequence on the gene. The adjacent DNA sequence unwinds and forms a DNA-sgRNA complex (R loop). The HNH and RuvC domains of the Cas9 protein cut the complementary and non-complementary strands of the sgRNA, respectively, to generate double-strand breaks (DSBs). These breaks are then repaired through non-homologous end joining (NHEJ) or homology-directed repair (HDR) (Szczelkun et al., 2014).

PAM is essential for the Cas9 protein to recognize and cleave target genes. However, the specific PAM sites recognized by Cas9 proteins vary among different sources. For instance, SaCas9 from *Staphylococcus aureus* recognizes NNGRRT (where N can be any nucleotide and R can be either A or G) (Ran et al., 2015), NmeCas9 from *Neisseria meningitidis* recognizes NNNNGATT (Zhang et al., 2015), and SpCas9 from *Streptococcus pyogenes* recognizes the simpler PAM sequence NGG (Anders et al., 2014). However, the requirement for Cas9 to recognize specific PAM sites during gene editing not only limits the locations where editing can be performed but also affects the efficiency and flexibility of the editing process. To address these limitations, scientists have developed various Cas9 variant systems that can extend PAM compatibility, such as xCas9, SpCas9-NG, SpG, and more (Hu et al., 2018; Nishimasu et al., 2018; Miller et al., 2020; Walton et al., 2020). One of the most recent advances is a “PAMless” Cas9 mutant protein called SpRY, which significantly increases the editable range of Cas9 and can identify practically all PAM sequences (Walton et al., 2020).

### 2.2 CRISPR-Cas12 system

**Figure 4:**
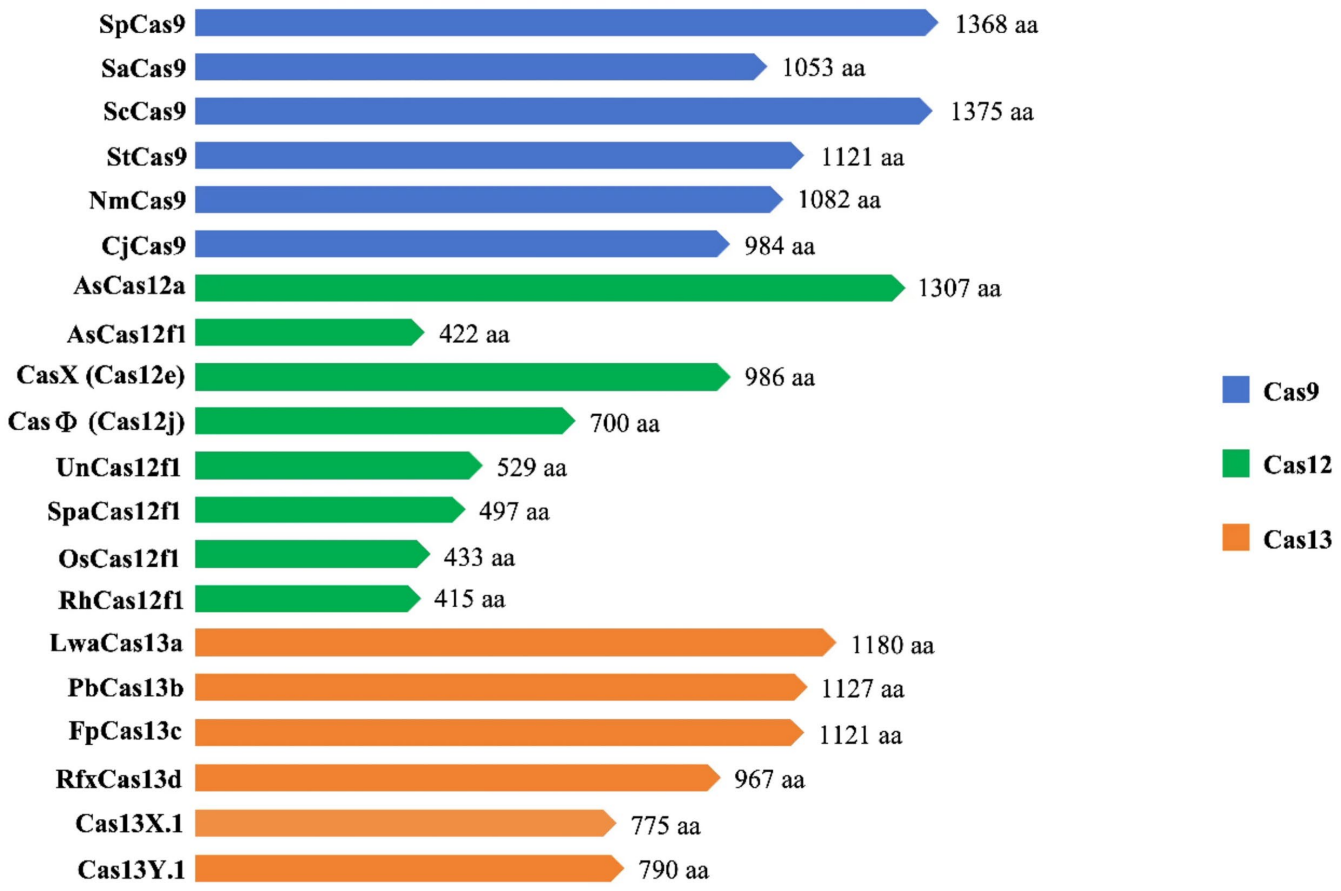
Cas9, Cas12 and Cas13 protein types and sizes.

Cas12 and Cas9 are the two commonly used CRISPR-Cas systems. However, Cas12 is generally smaller in size compared to Cas9 (Figure 4). During gene editing, CRISPR-Cas12 can recognize double-stranded DNA (dsDNA) rich in thymine (T) PAM sites only under the leadership of crRNA, and then accurately cut the desired sequence. Additionally, Cas12 also has target-specific trans-cleavage activity, allowing it to non-specifically cleave single-strand DNA (ssDNA) (Li et al., 2018) (Figure 3B). Following the identification of Cas12a (Cpf1) (Zetsche et al., 2015), scientists have successively developed several miniaturized Cas proteins such as Cas12b (C2c1) (Ming et al., 2020), Cas12e (CasX) (Liu J. J. et al., 2019), Cas12i (Yan et al., 2019), Cas12j (CasΦ) (Pausch et al., 2020), Cas12l (Sun et al., 2023), Cas12f (Cas14) (Harrington et al., 2018; Karvelis et al., 2020; Wu et al., 2021; Kim et al., 2022), among which the CRISPR-Cas12 genome editing technology has been advanced rapidly.

It is worth noting that Cas12f, originally classified as Cas14, was previously thought to only function as an endonuclease capable of specifically cutting desire ssDNA and other ssDNA non-specifically independent of PAM sequences (Harrington et al., 2018) (Figures 3D1,D2). Nevertheless, recent research has shown that Cas12f also has PAM-dependent targeting activity towards dsDNA, which differs from previous studies (Karvelis et al., 2020). Compared to the Cas9 protein, the wild-type Cas12 protein has lower gene editing efficiency and is more likely to produce deletion products (Xin et al., 2022). Recently, an engineered AsCas12f system known as “enAsCas12f” has been designed to increase the editing efficiency of the Cas12 protein. This system has a cleavage activity of up to 11.3 times that of the wild-type AsCas12f, although it is only a third as big as SpCas9. Due to this, it is among the most effective and small-sized CRISPR systems available, introducing a new area of CRISPR-based gene editing (Wu et al., 2023).

### 2.3 CRISPR-Cas13 system

Cas9 and Cas12 are enzymes that have the capability of modifying or editing DNA. In recent years, a new class of endonucleases called Cas13, which function to cleave RNA, has been discovered through extensive research (Abudayyeh et al., 2016). As shown in Figure 3C, Cas13 recognizes single-strand RNA (ssRNA) under the guidance of crRNA. Unlike Cas9 and Cas12, Cas13 does not require a specific PAM site and has a preference for adenine, cytosine, or uracil (A/C/U) located next to the protospacer flanking sites (PFS) (Abudayyeh et al., 2016). Six effector proteins belonging to the Cas13 family: Cas13a (C2c2) (Abudayyeh et al., 2016), Cas13b (Barrangou and Gersbach, 2017), Cas13c (Yan et al., 2018), Cas13d (Yan et al., 2018), Cas13X (Xu et al., 2021), and Cas13Y (Xu et al., 2021) have been identified by scientists. Among them, Cas13X is the smallest reported tool for editing RNA.

**Figure 5:**
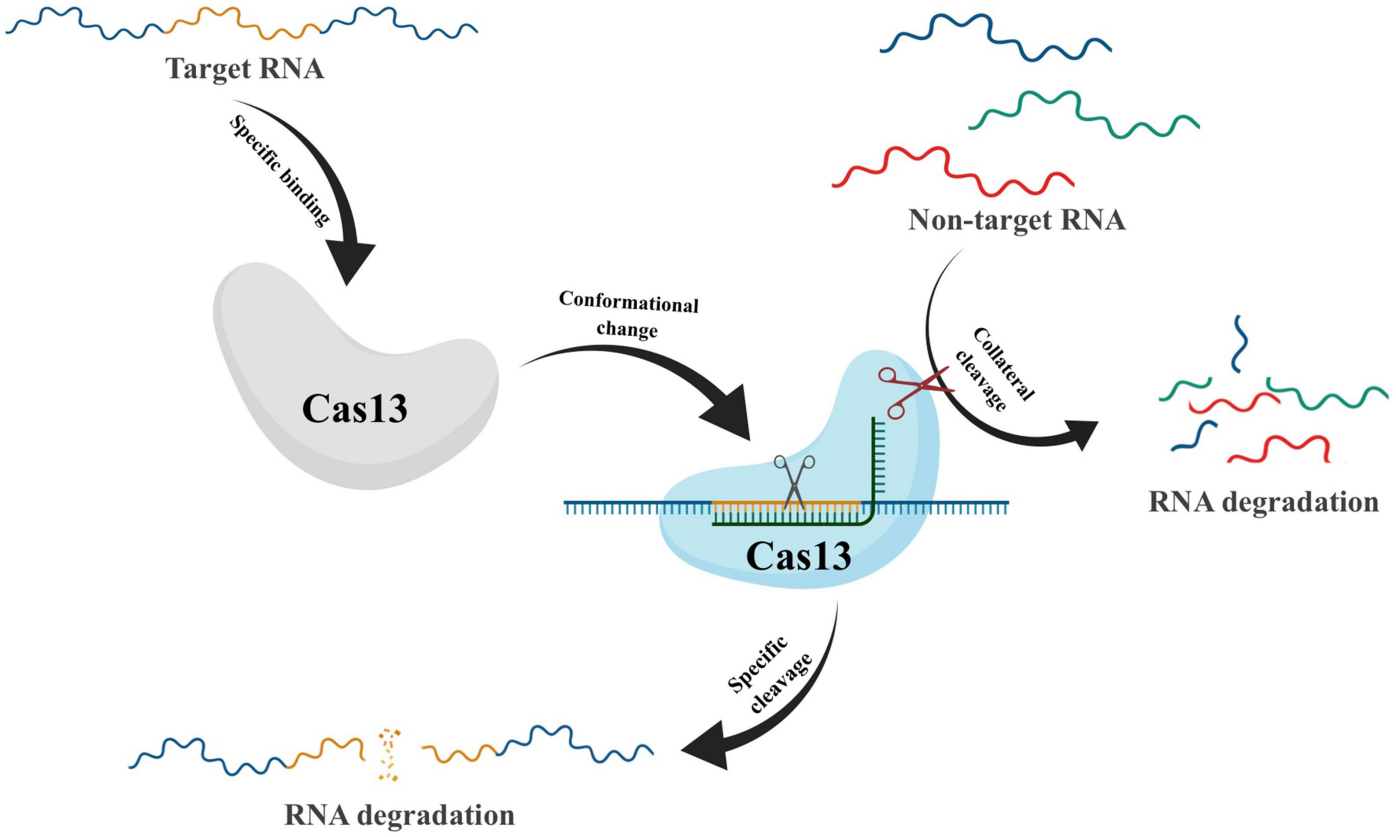
Schematic of Collateral Cleavage Phenomenon in CRISPR-Cas13 system. The Cas13-crRNA complex recognizes target RNA via base pairing with the cognate protospacer flanking sites (PFS) and cleaves the target RNA. In addition, binding of the target RNA by Cas13-crRNA activates a non-specific RNase activity which may lead to promiscuous cleavage of RNAs without complementarity to the crRNA guide sequence.

After crRNA-guided binding to target RNA, conformational changes in Cas13 frequently take place, which activate non-specific RNase activity. This leads to collateral cutting, a process whereby both target and non-target RNA are indiscriminately degraded (Figure 5) (Abudayyeh et al., 2016). This unique property has made CRISPR-Cas13 a promising technique for RNA detection (Gootenberg et al., 2017). However, the presence of collateral cleavage activity in Cas13 not only leads to severe off-target effects (Ai et al., 2022) but also causes disruption of the transcriptome, resulting in proliferation defects in target cells (Shi et al., 2023). These results have hindered the application of CRISPR-Cas13, prompting scientists to explore ways to reduce or eliminate the collateral cutting effect. In recent years, Yang et al. have conducted protein engineering, screening, and verification on Cas13X to develop high-fidelity Cas13 protein variants with high editing activity and minimal side cutting activity. This development is of great significance to the advancement of RNA gene editing technology (Tong et al., 2023). Compared to Cas9-mediated DNA editing technology, Cas13-based RNA editing tools target dynamically transcribed RNA without causing permanent changes to the genome. Additionally, the effects of RNA editing can be controlled through dose adjustments and other means, making it reversible and relatively safer.

## 3 CRISPR-Cas genome editing in filamentous fungi

In clinical treatments, the CRISPR-Cas system is typically delivered by adenovirus, adeno-associated virus, lentivirus, or other vectors with limited capacity. Therefore, the delivery process of Cas proteins with smaller volumes is more convenient (Xu et al., 2019). However, in the application of filamentous fungi, the delivery and expression of Cas proteins do not rely on viral vectors. As a result, researchers often choose the Cas9 and Cas12a systems, which have stronger editing capabilities, without considering the issue of Cas protein size. The Cas9 system has been well introduced into various filamentous fungi, including *Trichoderma reesei* (Liu et al., 2015), *Aspergillus fumigatus* (Zhang et al., 2016), *Aspergillus niger* (Zheng et al., 2019), *Aspergillus oryzae* (Katayama et al., 2019), *Cordyceps militaris* (Meng et al., 2022), *Beauveria bassiana* (Chen et al., 2017), etc. (Table 2). Since the Cas12a protein was discovered, filamentous fungi, including *Myceliophothora thermophile*, *Aspergillus nidulans*, and *Ashbaya gossypii,* have also been effectively utilized with the CRISPR-Cas12a system (Liu Q. et al., 2019; Vanegas et al., 2019; Jimenez et al., 2020; Roux et al., 2020). Nevertheless, there have not been any examples of filamentous fungi employing CRISPR-Cas13 as an RNA editing tool yet.

**Table 2:** Examples of the CRISPR-Cas system assisted in the editing of genes in filamentous fungi.

Species	Expression of Cas	Expression of sgRNA	Delivery methods	Screening marker	Editing methods	Target gene	Mutation efficiency	References
*A. fumigatus*	P*tef1*; T*cyc1*; Human codon-optimized Cas9	U6 promoter	PMT	*hphR, ble*	NHEJ	*pksP*	Single Gene: 25–53%	Fuller et al. (2015)
	P*gpdA*/P*niiA*; T*trpC*; Human codon-optimized Cas9	T7 *in vitro* transcription/U6 expression *in vivo*	PMT	*pyr4, hphR, egfp*	MMEJ	*pksP*, *cnaA*	Dual Gene: 95–100%	Zhang et al. (2016)
*A. niger*	P*glaA*; T*glaA*; codon-optimized Cas9	Endogenous 5SrRNA promoter	PMT	*hphR, amds*	HDR	*alba*, *pkac*, *fum5*, f*um1*	Single or multiple-gene: 33–100%	Zheng et al. (2019)
	P*tef*; T*tef*; Codon-optimized nCas9 fused to APOBEC1 to form BEC	U6 promoter from *A.oryzae*	PMT	–	BE	*pyrG*, *fwnA*, *prtT*, *hygR*, *pyrG*	Single Gene: 60% Dual Gene: 47–100%	Huang et al. (2019)
	P*pkiA*; T*glaA*; codon-optimized Cas9	tRNAPro1 promoter	PMT	*pyrG*	HDR	*xlnR*, *gaaR*	Single Gene: 80%	Kun et al. (2020)
*C. militaris*	P*cmgpd*;T*nos*; codon-optimized Cas9	T7 *in vitro* transcription	AMT, PMT	*blpR, egfp, ura3*	NHEJ/HDR	*ura3*	Single Gene: 11.76%	Chen et al. (2018)
	P*gpd*;T*trpC*; codon-optimized Cas9	5SrRNA promoter; T6	AMT, PMT	*hygR, pyr4*	NHEJ/HDR	*cmwc-1*, *cmvvd*	Single Gene: 55–89%; Dual Gene: 10%	Meng et al. (2022)
	P*cmgpd*; T*pc*; codon-optimized Cas9	P*trpC* with endogenous tRNA-processed system; T*nos*	AMT	*blpR*	NHEJ/HDR	*ura3*, *cns1*, *egt1*	multiple-gene: 18–26%	Chen et al. (2022)
	RNP is assembled from Cas9 and sgRNA expressed *in vitro*		PMT	*egfp, hygR*	NHEJ/HDR	*cmhk1*	Single Gene: 100%	Choi et al. (2023)
*B. bassiana*	P*gpdA*; T*trpC*; codon-optimized Cas9	T7 *in vitro* transcription	AMT, PMT	*egfp, ura5, bar*	NHEJ/HDR	*ura5*, *egfp*, *bbmp1*	Single Gene: 100%; Dual Gene: 39%; Three Genes: 5%	Chen et al. (2017)
*thermophila*	P*tef1*; T*trpC*; Codon-optimized nCas9 fused to APOBEC1 to form BEC	U6 expression *in vivo*	PMT	*Neo, bar, G418*	BE	*amds*, *cre-1*, *mtclr-2*	Single Gene: 17–93%	Zhang et al. (2022)
	P*tef1*; T*tprC*; codon-optimized Cas9	Endogenous U6 promoter; 6 T terminator	AMT	*bar, neo, egfp*	NHEJ/HDR	*amds*, *cre1*, *res1*, *gh1*, *alp*	Single Gene: 90–95%; Dual Gene: 61–70%; Four Genes: 22%	Liu et al. (2017)
	P*tef1*; codon-optimized Cas12a		PMT	*bar, neo*			Single Gene: 10–90%; Three Genes: 0–41%; Four Genes: 0–22%	Liu Q. et al. (2019)
*M. oryzae*	RNP is assembled from Cas9 and sgRNA expressed *in vitro*		PMT	–	NHEJ	*sdh*	Single Gene: 20%	Li et al. (2019)
*A. oryzae*	P*Aotef1*, P*amyB*; T*amyB*; codon-optimized Cas9	Endogenous U6 promoter; 6 T terminator	PMT	*ptrA, egfp*	NHEJ/HDR	*wa*, *pyrG*, *yA*, *niaD*	multiple-gene: 55.6–100%	Katayama et al. (2019)
*A. gossypii*	P*tef1*;T*cyc1*; Human codon-optimized Cas9	Endogenous U6 promoter; 6 T terminator	Electroporation	*G418*	HDR	*ade2*, *a754*, *fmp27*	Single Gene: 44–85%	Jiménez et al. (2019)
	P*tsa1*; T*eno1*; codon-optimized LbCas12a	Endogenous U6 promoter; 6 T terminator	PMT	*G418*	HDR	*his3*, *ade*, *trp1*, *leu2*, *ura3*	Single Gene: 19–77%; multiple-gene:10–30%	Jimenez et al. (2020)
*F. fujikuroi*	P*gpdA* and *TtrpC* from *A. nidulans*; codon-optimized Cas9	Endogenous U6 or 5SrRNA promoter	PMT	*hphR*	NHEJ	*fcc1*, *ura3*, *ppt1*	U6: 37.5%; 5SrRNA: 79.2%	Shi et al. (2019)
*A. alternata*	P*eno1*; T*cyc1*; codon-optimized Cas9	Endogenous U6 promoter; T*eno1*	Electroporation	*nat*	NHEJ/HDR	*ade2*	–	Min et al. (2016)
	P*tef1*/P*eno1*; codon-optimized Cas9	Endogenous U6 promoter	Electroporation	*nat*	HDR	*cph1*, *egfp*	Single or Dual gene: 25–100%	Vyas et al. (2018)
*F. oysporum*	RNP is assembled from Cas9 and sgRNA expressed *in vitro*		PMT	*hphR, egfp*	NHEJ/HDR	*ura5*, *blk1*	Single Gene: 9.5–53.8%	Wang et al. (2018)
*A. nidulans*	P*tef1*; T*tef1*; codon-optimized ErCas12a	Type III promoter derived from *Aspergillus fumigatus*	PMT	*argB*; *mRFP*	NHEJ/HDR	*albA*	–	Vanegas et al. (2022)
	P*tef1*; T*tef1*; codon-optimized LbCas12a	tRNA promoter	PMT	*pyrG*	HDR	*yA, albA*	Single Gene: 80–100%	Vanegas et al. (2019)
*T. reesei*	P*pdc,* P*Kbh1*; T*pdc*; codon-optimized Cas9	T7 *in vitro* transcription	PMT	*hygR*; *ura5*	NHEJ/HDR	*ura5, lae1, vib1*	Single Gene: 93–100%; Dual Gene: 16–45%; Three Genes: 4.2%	Liu et al. (2015)
	RNP is assembled from Cas9 and sgRNA expressed *in vitro*		PMT	*ura5*	NHEJ/HDR	*ura5*	NHEJ: 0–43%; HDR: 57–100%	Zou et al. (2021)
*G. lucidum*	RNP is assembled from Cas9 and sgRNA expressed *in vitro*		PMT	*pyrG*	NHEJ	*pyrG*	Single Gene: 37–79%	Eom et al. (2023)
	*gpd* intron 1; codon-optimized Cas9	T7 *in vitro* transcription	PMT	*hygR*; *ura3*	HDR	*ura3*	Single Gene: 93.1–96.3%	Tu et al. (2021)
*Coprinopsis cinerea*	Pcc*ded1*; *gpd2* exon-intron; codon-optimized Cas9	Endogenous U6 promoter	PMT	*gfp*; *kmR*	HDR	*gfp*	Single Gene: 21%	Sugano et al. (2017)
*Flammulina filiformis*	P*gpd*; codon-optimized Cas9	Endogenous U6 promoter	PMT	*pyrG*	HDR	*pyrG*	–	Liu X. et al. (2022)
*L. edodes*	*Plegpd*; codon-optimized Cas9	Endogenous U6 promoter	PMT	*hygR*	HDR	*hd1*	–	Moon et al. (2021)
*P. ostreatus*	P*ccef3*; codon-optimized Cas9	U6 promoter derived from *Coprinopsis cinerea*	PMT	*pyrG*; *hphR*	HDR	*fcy1*; *pyrG*	Single Gene: 80–100%	Boontawon et al. (2021)

### 3.1 Expression of the CRISPR-Cas system

There are three main methods to express the CRISPR-Cas system in filamentous fungi, and the expression strategies of Cas9 and Cas12 are identical as well. The first method involves transforming a plasmid containing both the sgRNA transcription sequence and the Cas9 protein expression sequence (Wang et al., 2020). This allows for continuous expression of both the Cas protein and sgRNA in the fungi, increasing the likelihood of successful cleavage or targeting of the target DNA/RNA. However, this approach is dependent on utilizing promoters to express the Cas protein and sgRNA *in vivo*. The second method involves transferring the sgRNA to be transcribed *in vitro* into a strain expressing the Cas protein (Liu et al., 2015). This method has the benefit of not requiring *in vivo* promoters for sgRNA expression, but it comes with extra work and the danger of sgRNA degradation. A third strategy is to transform ribonucleoproteins (RNP) assembled from Cas and sgRNA into the cells (Jan et al., 2019; Li et al., 2019). This method is not limited by Cas9 translation or sgRNA transcription levels, unlike the previous methods, and can be used in filamentous fungi that lack a codon usage database and cannot be codon-optimized for the host. However, directly converting RNP into fungal protoplasts presents the problem of low efficiency, which to some extent limits its application in fungi. According to recently published studies, extending the period of incubation and adding the chemical reagent Triton X-100 can significantly improve RNP’s conversion performance (Wu et al., 2020).

#### 3.1.1 Expression of Cas protein

Fungi do not natively contain the Cas protein, and the CRISPR-Cas system was initially identified in bacteria and archaea. Thus, fungal genomes that express the Cas9 protein and are fungal codon-optimized are required in order to use the CRISPR-Cas9 system in fungi. Without this optimization, the expression efficiency of *cas* genes is low and may even fail to successfully produce Cas proteins (Liu et al., 2015; Sugano et al., 2017). For example, Liu et al. (2015) were unable to construct a functional CRISPR-Cas system using Cas9 optimized for human codon preferences but were successful after using *Trichoderma reesei* codon-optimized Cas9. Similarly, Sugano et al. (2017) used Cas9 genes optimized for codon preferences in *humans*, *Arabidopsis*, *Candida*, and *Basidiomycetes* codon preferences to construct a CRISPR-Cas9 system in the gray-covered parasol fungus and found that only the *Basidiomycete* codon-optimized Cas9 showed an editing efficiency of 10.5%. In addition, studies have shown that human codon-optimized Cas protein genes can also be expressed in filamentous fungi with similar codon preferences, such as *Aspergillus fumigatus* (Zhang et al., 2016) and *Ashbya gossypii* (Jiménez et al., 2019). Filamentous fungi are eukaryotes, meaning their DNA is primarily located in the nucleus and controls nuclear inheritance. In order to correctly localize the Cas protein within the fungal cells, a nuclear localization signal (NLS) was introduced to both sides of the *cas* gene (Nodvig et al., 2015). Numerous studies have fused the enhanced green fluorescent protein (eGFP) gene to the optimized Cas in order to demonstrate the production and location of the Cas protein. Fluorescence is an indicator that the Cas protein has been effectively produced in the host fungus (Chen et al., 2017, 2018). In the construction of CRISPR system plasmid expression vectors for filamentous fungi, commonly used constitutive promoters include the Transient Receptor Potential Canonical Channel (*trpC*), glyceraldehyde-3-phosphate dehydrogenase (*gpdA*), and translation elongation factor 1 (*tef1*) genes from *Aspergillus nidulans.* These genes are commonly used in the construction of CRISPR system plasmid expression vectors for filamentous fungi (Matsu-Ura et al., 2015; Nodvig et al., 2015; Kuivanen et al., 2017). Alternatively, some studies have utilized endogenous promoters from the specific host fungus to drive expression of the Cas protein (Sugano et al., 2017; Chen et al., 2018).

#### 3.1.2 Expression of SgRNA

There are two approaches for generating sgRNA: *in vivo* transcription and *in vitro* transcription. *In vivo* transcription involves the employ of type II or type III promoters, while *in vitro* transcription is typically driven by a T7 promoter (Chen et al., 2017; Wang et al., 2020). Generally, using RNA polymerase type III promoters to drive sgRNA *in vivo* expression is preferred in the CRISPR-Cas system, as sgRNA lacks a poly A tail and a cap structure. Among these promoters, the u6 promoter of RNA polymerase type III is the most frequently employed in filamentous fungi (Fuller et al., 2015; Min et al., 2016; Huang et al., 2019; Zhang et al., 2022). One study demonstrated that the editing performance of the 5S rRNA promoter was higher than that of the u6 promoter, with editing efficiencies of 37.5 and 79.2%, respectively, in editing the *fcc1* gene of *Fusarium fujikuroi* (Shi et al., 2019). The 5S rRNA promoter has also been successfully used to drive *in vivo* transcription of sgRNA in other filamentous fungi, including *Aspergillus niger* (Zheng et al., 2019) and *Cordyceps militaris* (Meng et al., 2022). In addition to the u6 and 5S rRNA promoters, Song et al. validated that 36 tRNA promoters can be used to express sgRNA in *Aspergillus niger* (Song et al., 2018). However, RNA polymerase type II promoters are more commonly used in eukaryotic organisms than type III promoters. Therefore, RNA polymerase type II promoters may also be used to drive sgRNA expression, but this method requires the introduction of hammerhead ribozymes (HH) at the 5′ end of sgRNA and the introduction of hepatitis D virus ribozymes (HDV) at the 3′ end. By self-cleaving the two ribozymes, modifications at both ends of the RNA are eliminated, preventing the loss of sgRNA targeting by sgRNA outside of the nucleus (Nodvig et al., 2015; Kujoth et al., 2018). This method of driving sgRNA expression by RNA polymerase type II promoter has successfully been used for the editing of genes in filamentous fungi, including *Aspergillus aculeatus* (Nodvig et al., 2015), *Alternaria alternata* (Wenderoth et al., 2017), and *Cordyceps militaris* (Chen et al., 2022). However, Chen et al. were unable to successfully edit the *ura3* gene of *Cordyceps militaris* using this method, so they switched to the method of *in vitro* expression of sgRNA. Similarly, when editing the *fcc1* gene of *Fusarium fujikuroi*, Shi et al. (2019) found that the method of using RNA polymerase type II promoter was not effective, but the method of using RNA polymerase type III promoter was able to achieve gene editing. When the host promoters are unclear, researchers often choose *in vitro* transcription, as seen in studies on *Aspergillus fumigatus* (Zhang et al., 2016), *Trichoderma reesei* (Liu et al., 2015) and *Beauveria bassiana* (Chen et al., 2017). In contrast, sgRNA expressed *in vitro* has the advantage of not relying on promoters *in vivo*, but sgRNA expressed *in vivo* is simpler to operate and more efficient for gene editing. Additionally, since the coding DNA sequence (CDS) of filamentous fungus is discontinuous and split between exons and introns, with only the exons being expressed during gene expression, it is crucial to target the exon area of the CDS section when constructing sgRNA.

### 3.2 Delivery of the CRISPR-Cas9 system and screening markers

*Agrobacterium*-mediated transformation (AMT) and polyethylene glycol-mediated transformation (PMT) are the two primary integration methods for the CRISPR-Cas system vectors, which are frequently employed for genome editing in filamentous fungi (AMT) (Song et al., 2019). By employing PEG induction to introduce foreign DNA or RNA fragments into protoplasts, PMT causes these fragments to randomly integrate within the fungal genome, creating multiple copies (Yu et al., 2014). On the flip side, AMT utilizes the expression of Virulence genes (*Vir*) on the *Agrobacterium* tumor-inducing (Ti) plasmid to transfer DNA (T-DNA) and exogenous DNA into the mycelium, spores, or protoplasts of filamentous fungi, resulting in single copy integration (Sayari et al., 2019). Additionally, electroporation has been successfully used to transform plasmid vectors carrying the Cas9 gene into *Alternaria alternata* (Min et al., 2016; Vyas et al., 2018).

The screening of transformants requires the use of available markers. However, the selection of markers for filamentous fungal screening is limited. Common types of markers include resistance marker genes, nutrient deficiency genes, and phenotype reporter genes. Resistance genes, such as the hygromycin B resistance gene (*hphR*/*hygR*), glufosinate resistance gene (*bar*), and geneticin resistance gene (*G418*), have been effectively employed to screen positive strains. For example, filamentous fungi selected for *hph*/*hyg* as a positive screening marker include *Aspergillus fumigatus* (Fuller et al., 2015), *Aspergillus niger* (Kuivanen et al., 2019), *Talaromyces atroroseus* (Nielsen et al., 2017), *Pochonia chlamydosporia* (Youssar et al., 2019), *Leptosphaeria biglobosa* (Darma et al., 2019), *Fusarium fujikuroi* (Shi et al., 2019), *Fusarium oxysporum* (Wang et al., 2018), etc. The *bar* selection includes *Cordyceps militaris* (Chen et al., 2018), *Thermobrachicum celere* (Liu et al., 2017), *Neurospora crassa* (Matsu-Ura et al., 2015), and *Pyricularia oryzae* (Arazoe et al., 2015). G418 antibiotics are used to screen for positive filamentous fungi, such as *Ashbya gossypii* (Jiménez et al., 2019), *Ustilaginoidea virens* (Liang et al., 2018), and *Phytophthora sojae* (Miao et al., 2018). In addition to resistance genes, the expression of trophic markers, such as the acetamidase encoding gene (*amds*) and orotidine-5′-phosphate decarboxylase gene (*ura3*), is also an effective means to screen for positive filamentous fungi strains. For example, scientists have expressed *amds* genes derived from *Aspergillus nidulans* in *Aspergillus niger* (Zheng et al., 2019) and *Penicillium chrysogenum* (Pohl et al., 2016), giving these filamentous fungi the capacity to survive on medium with acetamide as the only nitrogen source, while wild-type strains cannot grow. *Pyr4* gene deficient strains selected by 5-FOA were also used as receptor strains for gene expression, and the researchers established screening markers for uridine deficiency and applied them to filamentous fungi such as *Mucor circinelloides* (Nagy et al., 2019) and *Aspergillus fumigatus* (Zhang et al., 2016). Enhanced green fluorescent protein (EGFP) and red fluorescent protein (RFP) are excellent indicators for isolating filamentous fungi by fluorescence microscopy or flow cytometry (Nodvig et al., 2015). Some scientists evaluated Cas expression levels by constructing Cas with eGFP (Liu et al., 2017; Chen et al., 2018).

### 3.3 NHEJ and HDR-mediated gene knockout

In filamentous fungi, CRISPR-Cas editing of genes is primarily accomplished via the NHEJ and HDR pathways to repair double-strand breaks (DSBs) caused by Cas protein splicing. The NHEJ repair pathway can occur throughout the cell cycle, with a major role played by the *ku70* and *ku80* proteins in the G1 phase (Sfeir et al., 2015). HDR is typically active in the S or G2 phase of the cell cycle, as sister chromatids provide homologous donor DNA fragments at this stage (Sfeir et al., 2015). Without the presence of a donation template DNA, the DSB ends produced by Cas9 are primarily joined together through NHEJ, which can result in frameshift mutations due to random base insertion or deletion. This ultimately leads to target gene inactivation or mutation. NHEJ-mediated gene knockout is frequently employed in the initial stages of the CRISPR-Cas system’s utilization to disrupt endogenous marker genes and verify the system’s construction success. It can also be employed for gene inactivation to reveal gene function (Bauer et al., 2015), as demonstrated in studies on *Aspergillus fumigatus* (Fuller et al., 2015), *Magnaporthe oryzae* (Li et al., 2019), *Fusarium fujikuroi* (Shi et al., 2019).

However, the deletions or insertions introduced by NHEJ at the cleavage sites were random and did not result in the changes expected. In contrast, HDR is a more effective method because it provides artificial DNA repair templates, which enable the exact introduction of mutations or sequences at the target spot. The performance of HDR-mediated gene editing is influenced by factors such as the distance between the homology arm and the DSB, the size of the homology arm, and the type of donor DNA fragment used. For example, Dong et al. found that in their research on *Aspergillus niger*, the closer the homologous arm was to the double-strand break, the higher the gene editing efficiency. They designed three donor DNA fragments containing the resistance screening marker gene *hygB*, with homologous arms of the same size at both ends but at different distances from the DSB (0 kb, 1 kb, and 5 kb). The resulting *hygB* integration efficiencies were 80, 50, and 10%, respectively (Dong et al., 2019). Liu et al. effectively implemented a CRISPR-Cas9 system in *T. reesei* by means of the introduction of homologous arms with different lengths around the selectable marker. They demonstrated that the addition of a pair of 200-bp homology arms resulted in a homologous recombination frequency of approximately 93%, allowing for CRISPR-Cas9-mediated gene knockout in *T. reesei* (Liu et al., 2015). In addition to linear donor DNA, successful gene editing has also been achieved in *Aspergillus oryzae* (Katayama et al., 2019) and *Trichoderma reesei* (Liu Q. et al., 2019) using circular donor DNA. Compared to linear DNA donors, circular DNA donors have been found to be more efficient in gene editing, likely due to the reduced integration into filamentous fungi chromosomes without a DSB, resulting in a lower false positive rate during screening (Chen et al., 2017). In recent years, single-strand oligonucleotides have also been used as homologous recombination donors for CRISPR-Cas-mediated genome editing in filamentous fungi, and research has shown that even 60-mer single-strand oligonucleotides can accurately alter target genes in *Aspergillus niger* employing the CRISPR-Cas system (Kun et al., 2020).

### 3.4 Multiple editing

Some fungal traits and natural products are usually regulated by multiple genes, making it necessary to simultaneously knock out target genes in practical applications. Yet, the capacity of conventional techniques such as ZFN and TALEN to simultaneously knock out several genes is hampered. Whereas, by expressing several sgRNAs in filamentous fungi, CRISPR-Cas technology enables simpler multigene editing. There are three main strategies for expressing multiple sgRNAs in filamentous fungi. The first method is to introduce a vector carrying the Cas9 expression sequence into the host fungi through the AMT method. Following that, introduce several mature sgRNAs generated *in vitro* into the Cas9-positive cells through the PMT method (Liu et al., 2015). This method has been successfully used in *Aspergillus fumigatus* (Zhang et al., 2016), *Aspergillus niger* (Kuivanen et al., 2016), and *Beauveria bassiana* (Chen et al., 2017). The second method involves using the tRNA-spacer system to express multiple sgRNAs. The sgRNAs are concatenated and interspaced by 5′ and 3′ splice sites of a tRNA, which are recognized by RNase P and RNase Z. This allows for the release of mature sgRNAs through the action of these enzymes (Phizicky et al., 2010). Employing polymerase III promoters and tRNA spacers, Nodvig et al. (2018) designed a vector system that can deliver Cas9 and several sgRNAs. With this approach, two point mutations and one gene insertion have been introduced in a single transformation experiment with great success and excellent performance. Chen et al. (2022) have developed a marker-free CRISPR-Cas9-TRAMA genome editing system by utilizing an endogenous tRNA-processed element, allowing for several gene-specific editing as well as large synthetic cluster elimination in *Cordyceps militaris*. It is worth noting that a trancrRNA is not required for the single-RNA-guided nuclease Cas12a to function. The capacity of Cas12a to use specialized CRISPR arrays to encode two or more crRNAs in a single transcript gives it an essential benefit over Cas9. This contains multiple crRNAs separated by short direct repetitions, making it simple to multiplex the editing of genes and regulation (Liu Q. et al., 2019). The third method involves employing a linker sequence to bind two distinct sgRNA cassettes together so that they are expressed under the same promoter and terminator. However, research has revealed that this strategy is less effective at editing than driving distinct sgRNAs by independent promoters (Kearns et al., 2015).

**Figure 6:**
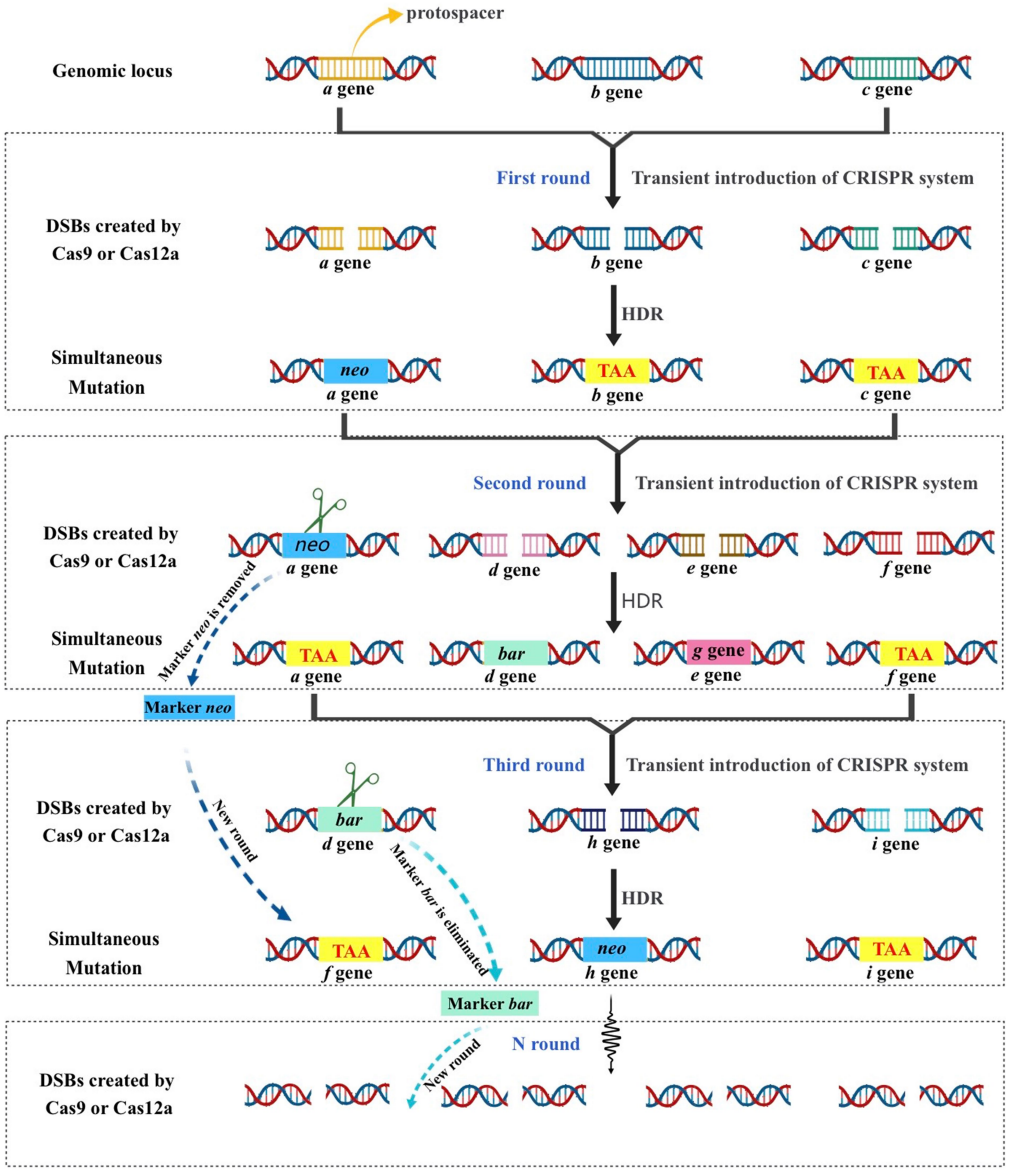
Schematic strategy of CRISPR–Cas-mediated marker recycling approach for iterative multiplex genome editing in *M. thermophila*. The hendecuple mutants were created through a series of three transformations, utilizing the selectable markers neo and bar. In the first transformation, a triple mutant was generated usingtransient CRISPR-Cas12a/Cas9-mediated homology-directed repair (HDR). This involved replacing an endogenous locus with the neo cassette and deleting two target genes without the use of markers. In the second transformation, the neo insert was removed and bar was used to replace another endogenous locus in the triple-mutant strain through HDR with the CRISPR-Cas12a/Cas9 system. Additionally, two other gene loci underwent seamless gene replacement and markerless gene disruption. For the third transformation, the octuple mutant was selected for genetic manipulation using the CRISPR system. The selectable marker neo was inserted into a new endogenous locus, while the marker bar was removed without leaving a scar, and another gene was deleted without the use of a marker. This allows the marker bar to be reused in subsequent transformations.

**Figure 7:**
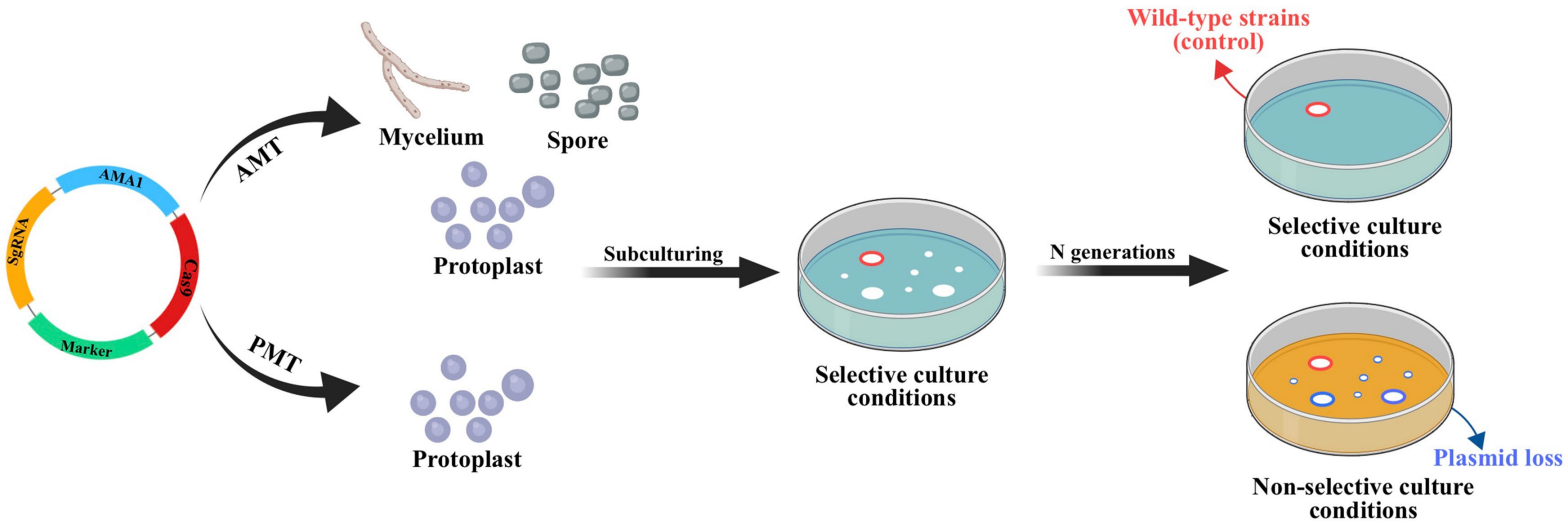
CRISPR-Cas system constructed based on autonomously replicating plasmids with MAM1 sequence. Leveraging the instability of autonomously replicating plasmid with an AMA1 sequence, the plasmid will be lost through subculture without resistance and the strain with lost plasmid no longer has the resistance gene of plasmid. This allows the resistance gene to be reused in subsequent transformations. Red circles indicate the transformant containing hyg obtained by ATMT; blue circles indicate wild-type strains (control).

The lack of available genetic markers is a major obstacle in the process of multigene editing in filamentous fungi. However, the development of CRISPR-Cas-assisted marker recycling technology (Camr technology) has reduced the need for multiple screening markers in experiments. An example of this method is the construction of hendecuple mutants *M. thermophila*, achieved through three successive transformations using two selectable markers, *neo* and *bar* (Liu Q. et al., 2019). In the first round, a triple mutant was created using transient CRISPR-Cas12a/Cas9-mediated homology-directed repair (HDR) (Figure 6). The *neo* cassette replaced an endogenous locus, while two target genes were deleted without the use of markers. In the second round, the *neo* cassette was removed and the *bar* was inserted into a new endogenous locus through HDR using the CRISPR-Cas system. Additionally, markerless gene disruption and seamless gene substitution were also applied to two more gene loci. The octuple mutant was selected in the third round to undergo additional genetic modification through the CRISPR technique. The selectable marker *neo* was inserted into a new endogenous locus, while the marker *bar* was seamlessly removed without leaving a scar. Another new gene also underwent markerless deletion. This allows for the reuse of the marker *bar* in subsequent transformation rounds. Moreover, the use of autonomously replicating plasmids with MAM1 sequences allows for the repeated use of selectable marker genes during transformation (Figure 7). This is because genes on such plasmids are not easily integrated into fungal genomes, and the plasmids are easily lost under non-selective culture conditions (Katayama et al., 2019; Wang et al., 2019; Meng et al., 2022).

### 3.5 CRISPR-dCas system

Nuclease-deficient Cas9 (dCas9) is a protein produced by inserting the point mutations H841A and D10A into the nuclease domains of RuvC and HNH. As a consequence, a protein that was previously capable of cleaving DNA is now incapable of cutting DNA. This results in a protein that retains DNA binding activity but can no longer cleave DNA (Qi et al., 2013, 2021). dCas9 can be engineered as a programmable transcription repressor by preventing the binding of RNA polymerase (RNAP) to promoter sequences, or as a transcription terminator by blocking the running RNAP (Bikard et al., 2013). CRISPR-dCas9 systems can be used for CRISPR-mediated transcriptional activation (CRISPRa) or CRISPR-mediated transcriptional interference (CRISPRi) of target genes by fusing dCas9 with transcriptional activators or repressors, directly manipulating the transcription process without altering the DNA sequence (Cano-Rodriguez et al., 2016; Mahas et al., 2018). dCas9 can also regulate chromatin state and gene expression by fusing with different epigenetic modifiers, allowing for exploration of the interaction among the epigenome, regulatory elements, and gene expression (Keung et al., 2014; Kearns et al., 2015). Epigenetic modifiers have been fused to dCas9 and applied to mammalian cells, such as histone acetyltransferase (HAT) (Hilton et al., 2015), human lysine specific demethylase 1 (LSD1) (Kearns et al., 2015), disruptor of telomeric silencing 1-like (DOT1L) (Cano-Rodriguez et al., 2016), and DNA methyltransferases (Dnmt) (Vojta et al., 2016), have been fused to dCas9 and applied to mammalian cells. Additionally, the application of CRISPR-dCas9-mediated epigenetic modification systems to target gene regulation in *Myxobacter* (Peng et al., 2018), *Malignant protozoa* (Xiao et al., 2019), *Arabidopsis thaliana* (Lee et al., 2019). Under typical laboratory culture conditions, the poor expression of most biosynthetic gene clusters (BGCs) limits the entire metabolic potential stored in the genomes of fungi. The identification of bioactive secondary metabolites driven by genomics may be sped up by the CRISPRa of fungal BGCs. The CRISPRa of fungal BGCs could accelerate genomics-driven bioactive secondary metabolite discovery. In 2020, the first CRISPRa system for filamentous fungi was formed by fusing dCas12a with a transcription activator, VP64-p65-Rta (VPR). This system has been shown to successfully activate a fluorescent reporter in *Aspergillus nidulans* and can also target the native nonribosomal peptide synthetase-like (NRPS-like) gene *micA* in both chromosomal and episomal contexts, resulting in increased production of the compound microperfuranone. Furthermore, multigene CRISPRa led to the discovery of the *mic* cluster product, dehydromicroperfuranone (Roux et al., 2020). Li et al. (2021) successfully applied CRISPR/dCas9-based histone epigenetic modification systems in *A. niger* to regulate the expression of secondary metabolic genes *breF*, *fuml*, and *fwnA*.

### 3.6 Base editors

There is no denying the fact that CRISPR-Cas systems, which introduce DSB for gene editing at target genes, may have a negative impact on genome stability (Doudna and Charpentier, 2014). In contrast, base editors are considered to be more reliable and safer gene editing tools as they can efficiently and accurately edit target genes without generating DSBs (Figure 3E) (Lei et al., 2021). Base editors consist of a catalytically inactive form of Cas9 (dcas9) or a Cas9 nickase mutant (nCas9), cytidine deaminases, including apolipoprotein B mRNA-editing enzyme catalytic subunit 1 (APOBEC1) and activation-induced cytidine deaminase (AID), as well as a uracil glycosylase inhibitor (UG1) (Komor et al., 2016; Nishida et al., 2016). Depending on the type of single base substitution achieved by the deaminases, base editors can be divided into adenine base editors (ABEs) and cytosine base editors (CBEs). ABEs can directly convert A to G, while CBEs can convert C to T. For example, ABEs can disrupt gene transcription by converting the promoter codon ATG to ACG, while CBEs can also convert codons such as CAA, CAG, and CGA to termination codons TAA, TAG, and TGA, respectively, to prematurely terminate gene expression (Komor et al., 2016). These base editors can be used for gene inactivation, gene function research, and targeted modification of biological metabolism, and are gradually being applied to animals, plants, bacteria, yeast, and filamentous fungi. In 2019, scientists successfully applied this technology to gene editing in *Aspergillus niger* for the first time. They used a cytosine base editor fused with nCas9 and rat cytosine deaminase (CD) rAPOBEC1 to convert cytosine in the target sequence recognized by CRISPR-Cas into thymine, showing good editing efficiency in the genes *pyrG*, *fwnA*, and *prtT* (Huang et al., 2019). Zhang et al. (2022) developed three cytosine base editors (Mtevo-BE4max, MtGAM-BE4max, and Mtevo-CDA1) in the thermophilic fungus *Myceliophthora thermophila* and effectively silenced the *amds*, *cre-1*, and *Mtclr-2* genes by accurately converting three codons (CAA, CAG, and CGA) to stop codons (TAA, TAG, or TGA). Among these editors, the Mtevo-CDA1 editor has an editing efficiency of up to 92.6%, making it a more suitable tool for cytosine base editing in *Myceliophthora thermophila*.

### 3.7 Prime editors

Prime editing (PE), a novel precision gene editing technique created in recent years by Liu et al. (Anzalone et al., 2019), enables accurate insertion and deletion of numerous bases without the use of DNA templates. The PE system is based on the CRISPR/Cas9 system and consists of two components (Figure 3F): prime editing guide RNA (pegRNA) and a fusion protein (Anzalone et al., 2019). PegRNA is a modified version of sgRNA with an additional RNA sequence at the 3′ end. This sequence can attach to reverse transcriptase (RT) and perform point mutations or insertion–deletion mutations as intended, acting as both a primer binding site (PBS) and a reverse transcriptase template (RTT) (Chen et al., 2021). The fusion protein is a novel protein created by combining nCas9 (HNH mutant) with reverse transcriptase. Under the guidance of pegRNA, the nCas9 nicking enzyme cuts the target DNA strand at the PAM site, and the broken strand then binds to the PBS sequence at the 3′ end of pegRNA. This initiates a reverse transcription reaction using the RT template sequence and reverse transcriptase (Anzalone et al., 2019). At the end of the reaction, a dynamic equilibrium is formed with 5′- and 3′-flap structures at the nick in the DNA strand. The 5′-end is then excised by the FEN1 protein, which has both 5′-endonuclease and exonuclease activity, and the 3′-end is edited by reverse transcriptase (Anzalone et al., 2019). After DNA ligation and repair, precise gene editing is achieved at the target site. However, Prime Editing has not yet been documented in filamentous fungi. While it has been successfully used in animals (Banskota et al., 2022; An et al., 2024) and plants (Li et al., 2020; Lin et al., 2020).

## 4 Conclusion and perspectives

The CRISPR-Cas system has been increasingly used for a growing number of model or non-model filamentous fungi in recent years. It plays an irreplaceable role in molecular breeding, metabolic regulation, and the strain advancement of filamentous fungi. The enzymes, organic acids, and secondary metabolites produced by filamentous fungi have been widely used in various areas, such as industries, food, and medicine. Scientists are promoting the development and application of filamentous fungi through CRISPR-Cas technology to decipher natural product biosynthesis pathways, enhance target natural product expression, and weaken or eliminate toxic products. For example, Liu et al. (2020) were able to eliminate the 15-kb citrinin biosynthesis gene cluster in Monascus purpureus industrial strain KL-001 by the CRISPR-Cas system. The poisonous citrinin was clearly removed, and the resulting homokaryotic mutants were stable. Additionally, there was a 2–5% rise in the pigment Monascus Red production. Utilizing the well-established CRISPR-Cas9 systems in *F. fujikuroi*, gibberellic acids’ (GAs) metabolic pathways were successfully rearranged by Shi et al. (2019) Gibberellic acids’ (GAs) metabolic pathways were successfully rearranged by Shi et al. (2019) to alter the product profile of GAs from primarily GA3 to a specially blended mixture of GA4 and GA7. Compared to the starting strain, the GA4/GA7 mixture’s ultimate production was enhanced by 8.1 times. In comparison to the progenitor controls, Zhang et al. (2020) detected a 2.17-fold increase in citric acid synthesis after using CRISPR-Cas9 to disrupt the genes encoding the orotidine-5′-decarboxylase (*pyrG*). The utilization of CRISPR-Cas9-mediated gene deletion technology in *A. niger* for a metabolic engineering application was proven by Kuivanen et al. (2016). They produced a strain that allows D-galacturonic acid to be efficiently converted to galactaric acid.

This article provides an introduction to the principle, function, and classification of CRISPR-Cas, as well as its application strategies and research progress in filamentous fungi. It aims to assist readers in selecting the appropriate CRISPR-Cas system for their specific needs. Scientists typically choose Cas9 or Cas12a, which have stronger editing abilities, to modify the genes of filamentous fungi (Xin et al., 2022). The natural spCas9 is a popular choice among researchers due to its powerful editing efficiency and ability to recognize relatively simple PAM sequences (Anders et al., 2014). In recent years, scientists have developed Cas mutants such as SpRY, enAsCas12f, dCas9, and nCas9 to overcome the limitations of wild-type Cas proteins (Walton et al., 2020; Li et al., 2021; Chen et al., 2022; Zhang et al., 2022; Wu et al., 2023). When constructing the CRISPR-Cas system in filamentous fungi, it is important to choose an appropriate expression strategy based on the specific situation. ① For filamentous fungi that lack a codon usage database and cannot be codon optimized for the host, the strategy of transforming RNPs assembled from Cas and sgRNA synthesized *in vitro* should be prioritized; ② Resistance genes can be reused by using autonomous replicating plasmids with the MAM1 sequence or CRISPR-Cas-assisted marker recycling technology; ③ The targeting efficiency of gRNA in the complex intracellular physiological and biochemical environments of vivo can affect the editing efficiency of CRISPR, therefore, it’s a well-suited option to create numerous sgRNAs for synchronous editing at a target gene to enhance the editing efficiency.

The forms of gene editing in filamentous fungi mainly include deletion, insertion, transcriptional interference or activation of specific sequences, and base editors, which are based on the CRISPR-Cas system (Huang et al., 2019; Kuivanen et al., 2019; Li et al., 2021; Zhang et al., 2022). Compared to traditional gene editing methods, CRISPR-dCas technology allows for the regulation of target gene transcription without altering DNA sequences (Mahas et al., 2018). It is also effective in precisely editing target genes without causing double-strand breaks (Lei et al., 2021). While CRISPR-dCas technology and BEs have been most frequently used in animals, bacteria, and other cells, they are not yet commonly used in filamentous fungi. However, they are expected to become the future trend and a safer option for gene editing in these fungi due to their unique advantages.

## Author contributions

QS: Writing – original draft, Writing – review & editing, Conceptualization, Investigation, Methodology, Software. HR: Writing – review & editing, Supervision. HZ: Software, Supervision, Writing – review & editing. TW: Supervision, Writing – review & editing, Investigation. KZ: Writing – original draft. WH: Writing – original draft. RD: Writing – review & editing. TM: Writing – review & editing. HQ: Writing – review & editing. YZ: Writing – review & editing.

## References

[ref1] Abudayyeh O. O. Gootenberg J. S. Konermann S. Joung J. Slaymaker I. M. Cox D. B.. (2016). C2c2 is a single-component programmable RNA-guided RNA-targeting CRISPR effector. Science 353:aaf5573. doi: 10.1126/science.aaf5573 27256883 PMC5127784

[ref2] Ai Y. Liang D. Wilusz J. E. (2022). CRISPR/Cas13 effectors have differing extents of off-target effects that limit their utility in eukaryotic cells. Nucleic Acids Res. 50:e65. doi: 10.1093/nar/gkac159, PMID: 35244715 PMC9226543

[ref3] An M. Raguram A. Du S. W. Banskota S. Davis J. R. Newby G. A.. (2024). Engineered virus-like particles for transient delivery of prime editor ribonucleoprotein complexes in vivo. Nat. Biotechnol. doi: 10.1038/s41587-023-02078-y, PMID: 38191664 PMC11228131

[ref4] Anders C. Niewoehner O. Duerst A. Jinek M. (2014). Structural basis of PAM-dependent target DNA recognition by the Cas9 endonuclease. Nature 513, 569–573. doi: 10.1038/nature13579, PMID: 25079318 PMC4176945

[ref5] Anzalone A. V. Randolph P. B. Davis J. R. Sousa A. A. Koblan L. W. Levy J. M.. (2019). Search-and-replace genome editing without double-strand breaks or donor DNA. Nature 576, 149–157. doi: 10.1038/s41586-019-1711-4, PMID: 31634902 PMC6907074

[ref6] Arazoe T. Ogawa T. Miyoshi K. Yamato T. Ohsato S. Sakuma T.. (2015). Tailor-made TALEN system for highly efficient targeted gene replacement in the rice blast fungus. Biotechnol. Bioeng. 112, 1335–1342. doi: 10.1002/bit.25559, PMID: 25683503

[ref7] Ashraf S. A. Elkhalifa A. Siddiqui A. J. Patel M. Awadelkareem A. M. Snoussi M.. (2020). Cordycepin for health and wellbeing: a potent bioactive metabolite of an entomopathogenic cordyceps medicinal fungus and its nutraceutical and therapeutic potential. Molecules 25:2735. doi: 10.3390/molecules25122735, PMID: 32545666 PMC7356751

[ref8] Banskota S. Raguram A. Suh S. Du S. W. Davis J. R. Choi E. H.. (2022). Engineered virus-like particles for efficient in vivo delivery of therapeutic proteins. Cell 185, 250–265. doi: 10.1016/j.cell.2021.12.021, PMID: 35021064 PMC8809250

[ref9] Barrangou R. Gersbach C. A. (2017). Expanding the CRISPR toolbox: targeting RNA with Cas13b. Mol. Cell 65, 582–584. doi: 10.1016/j.molcel.2017.02.002, PMID: 28212745

[ref10] Bauer D. E. Canver M. C. Orkin S. H. (2015). Generation of genomic deletions in mammalian cell lines via CRISPR/Cas9. J. Vis. Exp. 95:e52118. doi: 10.3791/52118 PMC4279820 25549070

[ref11] Bikard D. Jiang W. Samai P. Hochschild A. Zhang F. Marraffini L. A. (2013). Programmable repression and activation of bacterial gene expression using an engineered CRISPR-Cas system. Nucleic Acids Res. 41, 7429–7437. doi: 10.1093/nar/gkt520, PMID: 23761437 PMC3753641

[ref12] Boontawon T. Nakazawa T. Inoue C. Osakabe K. Kawauchi M. Sakamoto M.. (2021). Efficient genome editing with CRISPR/Cas9 in pleurotus ostreatus. AMB Express 11:30. doi: 10.1186/s13568-021-01193-w 33609205 PMC7897337

[ref13] Cano-Rodriguez D. Gjaltema R. A. Jilderda L. J. Jellema P. Dokter-Fokkens J. Ruiters M. H.. (2016). Writing of H3K4Me3 overcomes epigenetic silencing in a sustained but context-dependent manner. Nat. Commun. 7:12284. doi: 10.1038/ncomms12284 27506838 PMC4987519

[ref14] Carter J. Wiedenheft B. (2015). Snapshot: CRISPR-RNA-guided adaptive immune systems. Cell 163:260. doi: 10.1016/j.cell.2015.09.011, PMID: 26406380 PMC4668126

[ref15] Chen P. J. Hussmann J. A. Yan J. Knipping F. Ravisankar P. Chen P. F.. (2021). Enhanced prime editing systems by manipulating cellular determinants of editing outcomes. Cell 184, 5635–5652. doi: 10.1016/j.cell.2021.09.018, PMID: 34653350 PMC8584034

[ref16] Chen J. Lai Y. Wang L. Zhai S. Zou G. Zhou Z.. (2017). CRISPR/Cas9-mediated efficient genome editing via blastospore-based transformation in entomopathogenic fungus Beauveria bassiana. Sci. Rep. 8:45763. doi: 10.1038/srep45763, PMID: 28368054 PMC5377935

[ref17] Chen B. X. Wei T. Ye Z. W. Yun F. Kang L. Z. Tang H. B.. (2018). Efficient CRISPR-Cas9 gene disruption system in edible-medicinal mushroom cordyceps militaris. Front. Microbiol. 9:1157. doi: 10.3389/fmicb.2018.01157 29946301 PMC6005869

[ref18] Chen B. X. Xue L. N. Wei T. Wang N. Zhong J. R. Ye Z. W.. (2022). Multiplex gene precise editing and large DNA fragment deletion by the CRISPR-Cas9-trama system in edible mushroom cordyceps militaris. Microb. Biotechnol. 15, 2982–2991. doi: 10.1111/1751-7915.14147, PMID: 36134724 PMC9733643

[ref19] Choi H. Park S. W. Oh J. Kim C. S. Sung G. H. Sang H. (2023). Efficient disruption of CmHK1 using CRISPR/Cas9 ribonucleoprotein delivery in cordyceps militaris. FEMS Microbiol. Lett. 370:fnad072. doi: 10.1093/femsle/fnad072, PMID: 37475654

[ref20] Darma R. Lutz A. Elliott C. E. Idnurm A. (2019). Identification of a gene cluster for the synthesis of the plant hormone abscisic acid in the plant pathogen leptosphaeria maculans. Fungal Genet. Biol. 130, 62–71. doi: 10.1016/j.fgb.2019.04.015, PMID: 31034868

[ref21] Dong H. Zheng J. Yu D. Wang B. Pan L. (2019). Efficient genome editing in Aspergillus niger with an improved recyclable CRISPR-HDR toolbox and its application in introducing multiple copies of heterologous genes. J. Microbiol. Methods 163:105655. doi: 10.1016/j.mimet.2019.105655, PMID: 31226337

[ref22] Doudna J. A. Charpentier E. (2014). Genome editing. The new frontier of genome engineering with CRISPR-Cas9. Science 346:1258096. doi: 10.1126/science.1258096 25430774

[ref23] Dusengemungu L. Kasali G. Gwanama C. Ouma K. O. (2020). Recent advances in biosorption of copper and cobalt by filamentous fungi. Front. Microbiol. 11:582016. doi: 10.3389/fmicb.2020.582016, PMID: 33408701 PMC7779407

[ref24] Eom H. Choi Y. J. Nandre R. Han H. G. Kim S. Kim M.. (2023). The Cas9-gRNA ribonucleoprotein complex-mediated editing of pyrG in Ganoderma lucidum and unexpected insertion of contaminated DNA fragments. Sci. Rep. 13:11133. doi: 10.1038/s41598-023-38331-2 37429890 PMC10333205

[ref25] Fuller K. K. Chen S. Loros J. J. Dunlap J. C. (2015). Development of the CRISPR/Cas9 system for targeted gene disruption in *Aspergillus fumigatus*. Eukaryot. Cell 14, 1073–1080. doi: 10.1128/EC.00107-15, PMID: 26318395 PMC4621320

[ref26] Gootenberg J. S. Abudayyeh O. O. Lee J. W. Essletzbichler P. Dy A. J. Joung J.. (2017). Nucleic acid detection with CRISPR-Cas13a/C2c2. Science 356, 438–442. doi: 10.1126/science.aam9321, PMID: 28408723 PMC5526198

[ref27] Gupta D. Bhattacharjee O. Mandal D. Sen M. K. Dey D. Dasgupta A.. (2019). CRISPR-Cas9 system: a new-fangled dawn in gene editing. Life Sci. 232:116636. doi: 10.1016/j.lfs.2019.116636, PMID: 31295471

[ref28] Guzmán-Chávez F. Zwahlen R. D. Bovenberg R. A. L. Driessen A. J. M. (2018). Engineering of the filamentous fungus penicillium chrysogenum as cell factory for natural products. Front. Microbiol. 9:2768. doi: 10.3389/fmicb.2018.02768, PMID: 30524395 PMC6262359

[ref29] Harrington L. B. Burstein D. Chen J. S. Paez-Espino D. Ma E. Witte I. P.. (2018). Programmed DNA destruction by miniature CRISPR-Cas14 enzymes. Science 362, 839–842. doi: 10.1126/science.aav4294, PMID: 30337455 PMC6659742

[ref30] Hilton I. B. D'Ippolito A. M. Vockley C. M. Thakore P. I. Crawford G. E. Reddy T. E.. (2015). Epigenome editing by a CRISPR-Cas9-based acetyltransferase activates genes from promoters and enhancers. Nat. Biotechnol. 33, 510–517. doi: 10.1038/nbt.3199, PMID: 25849900 PMC4430400

[ref31] Hsu P. D. Scott D. A. Weinstein J. A. Ran F. A. Konermann S. Agarwala V.. (2013). DNA targeting specificity of RNA-guided Cas9 nucleases. Nat. Biotechnol. 31, 827–832. doi: 10.1038/nbt.2647, PMID: 23873081 PMC3969858

[ref32] Hu J. H. Miller S. M. Geurts M. H. Tang W. Chen L. Sun N.. (2018). Evolved Cas9 variants with broad PAM compatibility and high DNA specificity. Nature 556, 57–63. doi: 10.1038/nature26155, PMID: 29512652 PMC5951633

[ref33] Huang L. Dong H. Zheng J. Wang B. Pan L. (2019). Highly efficient single base editing in Aspergillus niger with CRISPR/Cas9 cytidine deaminase fusion. Microbiol. Res. 223-225, 44–50. doi: 10.1016/j.micres.2019.03.007, PMID: 31178050

[ref34] Huang J. Zhou Y. Li J. Lu A. Liang C. (2022). CRISPR/Cas systems: delivery and application in gene therapy. Front. Bioeng. Biotechnol. 10:942325. doi: 10.3389/fbioe.2022.942325, PMID: 36483767 PMC9723151

[ref35] Jan V. P. Escobar N. Wosten H. Lugones L. G. Ohm R. A. (2019). High-throughput targeted gene deletion in the model mushroom schizophyllum commune using pre-assembled Cas9 ribonucleoproteins. Sci. Rep. 9:7632. doi: 10.1038/s41598-019-44133-2, PMID: 31113995 PMC6529522

[ref36] Jiang F. Doudna J. A. (2017). CRISPR-Cas9 structures and mechanisms. Annu. Rev. Biophys. 46, 505–529. doi: 10.1146/annurev-biophys-062215-010822, PMID: 28375731

[ref37] Jimenez A. Hoff B. Revuelta J. L. (2020). Multiplex genome editing in Ashbya gossypii using CRISPR-Cpf1. New Biotechnol. 57, 29–33. doi: 10.1016/j.nbt.2020.02.002, PMID: 32194155

[ref38] Jiménez A. Muñoz Fernández G. Ledesma Amaro R. Buey R. M. Revuelta J. L. (2019). One-vector CRISPR/Cas9 genome engineering of the industrial fungus Ashbya gossypii. Microb. Biotechnol. 12, 1293–1301. doi: 10.1111/1751-7915.13425, PMID: 31055883 PMC6801137

[ref39] Jinek M. Chylinski K. Fonfara I. Hauer M. Doudna J. A. Charpentier E. (2012). A programmable dual-RNA-guided DNA endonuclease in adaptive bacterial immunity. Science 337, 816–821. doi: 10.1126/science.1225829, PMID: 22745249 PMC6286148

[ref40] Karvelis T. Bigelyte G. Young J. K. Hou Z. Zedaveinyte R. Budre K.. (2020). PAM recognition by miniature CRISPR-Cas12f nucleases triggers programmable double-stranded DNA target cleavage. Nucleic Acids Res. 48, 5016–5023. doi: 10.1093/nar/gkaa208, PMID: 32246713 PMC7229846

[ref41] Katayama T. Nakamura H. Zhang Y. Pascal A. Fujii W. Maruyama J. I. (2019). Forced recycling of an AMA1-based genome-editing plasmid allows for efficient multiple gene deletion/integration in the industrial filamentous fungus Aspergillus oryzae. Appl. Environ. Microbiol. 85:e01896-18. doi: 10.1128/AEM.01896-18, PMID: 30478227 PMC6344613

[ref42] Kearns N. A. Pham H. Tabak B. Genga R. M. Silverstein N. J. Garber M.. (2015). Functional annotation of native enhancers with a Cas9-histone demethylase fusion. Nat. Methods 12, 401–403. doi: 10.1038/nmeth.3325 25775043 PMC4414811

[ref43] Keung A. J. Bashor C. J. Kiriakov S. Collins J. J. Khalil A. S. (2014). Using targeted chromatin regulators to engineer combinatorial and spatial transcriptional regulation. Cell 158, 110–120. doi: 10.1016/j.cell.2014.04.047, PMID: 24995982 PMC4110908

[ref44] Kim Y. G. Cha J. Chandrasegaran S. (1996). Hybrid restriction enzymes: zinc finger fusions to Fok I cleavage domain. Proc. Natl. Acad. Sci. USA 93, 1156–1160. doi: 10.1073/pnas.93.3.1156, PMID: 8577732 PMC40048

[ref45] Kim D. Y. Lee J. M. Moon S. B. Chin H. J. Park S. Lim Y.. (2022). Efficient CRISPR editing with a hypercompact Cas12f1 and engineered guide RNAs delivered by adeno-associated virus. Nat. Biotechnol. 40, 94–102. doi: 10.1038/s41587-021-01009-z, PMID: 34475560 PMC8763643

[ref46] Komor A. C. Kim Y. B. Packer M. S. Zuris J. A. Liu D. R. (2016). Programmable editing of a target base in genomic DNA without double-stranded DNA cleavage. Nature 533, 420–424. doi: 10.1038/nature17946, PMID: 27096365 PMC4873371

[ref47] Kuivanen J. Arvas M. Richard P. (2017). Clustered genes encoding 2-keto-l-gulonate reductase and l-idonate 5-dehydrogenase in the novel fungal d-glucuronic acid pathway. Front. Microbiol. 8:225. doi: 10.3389/fmicb.2017.00225, PMID: 28261181 PMC5306355

[ref48] Kuivanen J. Korja V. Holmstrom S. Richard P. (2019). Development of microtiter plate scale CRISPR/Cas9 transformation method for Aspergillus niger based on in vitro assembled ribonucleoprotein complexes. Fungal Biol Biotechnol. 6:3. doi: 10.1186/s40694-019-0066-9 30923622 PMC6419801

[ref49] Kuivanen J. Wang Y. J. Richard P. (2016). Engineering Aspergillus niger for galactaric acid production: elimination of galactaric acid catabolism by using RNA sequencing and CRISPR/Cas9. Microb. Cell Factories 15:210. doi: 10.1186/s12934-016-0613-5 PMC5153877 27955649

[ref50] Kujoth G. C. Sullivan T. D. Merkhofer R. Lee T. J. Wang H. Brandhorst T.. (2018). CRISPR/CAS9-mediated gene disruption reveals the importance of zinc metabolism for fitness of the dimorphic fungal pathogen blastomyces dermatitidis. MBio 9, e412–e418. doi: 10.1128/mBio.00412-18 PMC5885028 29615501

[ref51] Kun R. S. Meng J. Salazar-Cerezo S. Makela M. R. de Vries R. P. Garrigues S. (2020). CRISPR/CAS9 facilitates rapid generation of constitutive forms of transcription factors in Aspergillus niger through specific on-site genomic mutations resulting in increased saccharification of plant biomass. Enzym. Microb. Technol. 136:109508. doi: 10.1016/j.enzmictec.2020.109508, PMID: 32331715

[ref52] Lee J. E. Neumann M. Duro D. I. Schmid M. (2019). CRISPR-based tools for targeted transcriptional and epigenetic regulation in plants. PLoS One 14:e222778. doi: 10.1371/journal.pone.0222778, PMID: 31557222 PMC6762090

[ref53] Lei Z. Meng H. Lv Z. Liu M. Zhao H. Wu H.. (2021). Detect-seq reveals out-of-protospacer editing and target-strand editing by cytosine base editors. Nat. Methods 18, 643–651. doi: 10.1038/s41592-021-01172-w, PMID: 34099937

[ref54] Li S. Y. Cheng Q. X. Liu J. K. Nie X. Q. Zhao G. P. Wang J. (2018). CRISPR-Cas12a has both cis- and trans-cleavage activities on single-stranded DNA. Cell Res. 28, 491–493. doi: 10.1038/s41422-018-0022-x, PMID: 29531313 PMC5939048

[ref55] Li X. Huang L. Pan L. Wang B. Pan L. (2021). CRISPR/dCas9-mediated epigenetic modification reveals differential regulation of histone acetylation on Aspergillus niger secondary metabolite. Microbiol. Res. 245:126694. doi: 10.1016/j.micres.2020.126694, PMID: 33482403

[ref56] Li H. Li J. Chen J. Yan L. Xia L. (2020). Precise modifications of both exogenous and endogenous genes in rice by prime editing. Mol. Plant 13, 671–674. doi: 10.1016/j.molp.2020.03.011, PMID: 32222486

[ref57] Li S. Song Z. Liu C. Chen X. L. Han H. (2019). Biomimetic mineralization-based CRISPR/Cas9 ribonucleoprotein nanoparticles for gene editing. ACS Appl. Mater. Interfaces 11, 47762–47770. doi: 10.1021/acsami.9b17598, PMID: 31773942

[ref58] Liang Y. Han Y. Wang C. Jiang C. Xu J. R. (2018). Targeted deletion of the USTA and UvSLT2 genes efficiently in Ustilaginoidea virens with the CRISPR-CAS9 system. Front. Plant Sci. 9:699. doi: 10.3389/fpls.2018.00699 29881395 PMC5976777

[ref59] Lin Q. Zong Y. Xue C. Wang S. Jin S. Zhu Z.. (2020). Prime genome editing in rice and wheat. Nat. Biotechnol. 38, 582–585. doi: 10.1038/s41587-020-0455-x, PMID: 32393904

[ref60] Liu W. An C. Shu X. Meng X. Yao Y. Zhang J.. (2020). A dual-plasmid CRISPR/Cas system for mycotoxin elimination in polykaryotic industrial fungi. ACS Synth. Biol. 9, 2087–2095. doi: 10.1021/acssynbio.0c00178, PMID: 32531165

[ref61] Liu R. Chen L. Jiang Y. Zhou Z. Zou G. (2015). Efficient genome editing in filamentous fungus Trichoderma reesei using the CRISPR/Cas9 system. Cell Discov. 1:15007. doi: 10.1038/celldisc.2015.7 27462408 PMC4860831

[ref62] Liu L. Chen Z. Liu W. Ke X. Tian X. Chu J. (2022). Cephalosporin C biosynthesis and fermentation in Acremonium chrysogenum. Appl. Microbiol. Biotechnol. 106, 6413–6426. doi: 10.1007/s00253-022-12181-w, PMID: 36114850

[ref63] Liu X. Dong J. Liao J. Tian L. Qiu H. Wu T.. (2022). Establishment of CRISPR/Cas9 genome-editing system based on dual sgRNAs in Flammulina filiformis. J. Fungi. 8:693. doi: 10.3390/jof8070693, PMID: 35887449 PMC9318071

[ref64] Liu Q. Gao R. Li J. Lin L. Zhao J. Sun W.. (2017). Development of a genome-editing CRISPR/Cas9 system in thermophilic fungal myceliophthora species and its application to hyper-cellulase production strain engineering. Biotechnol. Biofuels 10:1. doi: 10.1186/s13068-016-0693-9 28053662 PMC5209885

[ref65] Liu J. J. Orlova N. Oakes B. L. Ma E. Spinner H. B. Baney K.. (2019). Casx enzymes comprise a distinct family of RNA-guided genome editors. Nature 566, 218–223. doi: 10.1038/s41586-019-0908-x, PMID: 30718774 PMC6662743

[ref66] Liu Q. Zhang Y. Li F. Li J. Sun W. Tian C. (2019). Upgrading of efficient and scalable CRISPR-Cas-mediated technology for genetic engineering in thermophilic fungus Myceliophthora thermophila. Biotechnol. Biofuels 12:293. doi: 10.1186/s13068-019-1637-y 31890021 PMC6927189

[ref67] Ma X. Zhu Q. Chen Y. Liu Y. G. (2016). CRISPR/Cas9 platforms for genome editing in plants: developments and applications. Mol. Plant 9, 961–974. doi: 10.1016/j.molp.2016.04.009, PMID: 27108381

[ref68] Mahas A. Neal S. C. J. Mahfouz M. M. (2018). Harnessing CRISPR/Cas systems for programmable transcriptional and post-transcriptional regulation. Biotechnol. Adv. 36, 295–310. doi: 10.1016/j.biotechadv.2017.11.008, PMID: 29197619

[ref69] Makarova K. S. Haft D. H. Barrangou R. Brouns S. J. Charpentier E. Horvath P.. (2011). Evolution and classification of the CRISPR-Cas systems. Nat. Rev. Microbiol. 9, 467–477. doi: 10.1038/nrmicro2577, PMID: 21552286 PMC3380444

[ref70] Makarova K. S. Wolf Y. I. Alkhnbashi O. S. Costa F. Shah S. A. Saunders S. J.. (2015). An updated evolutionary classification of CRISPR-Cas systems. Nat. Rev. Microbiol. 13, 722–736. doi: 10.1038/nrmicro3569, PMID: 26411297 PMC5426118

[ref71] Makarova K. S. Wolf Y. I. Iranzo J. Shmakov S. A. Alkhnbashi O. S. Brouns S.. (2020). Evolutionary classification of CRISPR-Cas systems: a burst of class 2 and derived variants. Nat. Rev. Microbiol. 18, 67–83. doi: 10.1038/s41579-019-0299-x, PMID: 31857715 PMC8905525

[ref72] Matsu-Ura T. Baek M. Kwon J. Hong C. (2015). Efficient gene editing in Neurospora crassa with CRISPR technology. Fungal Biol. Biotechnol. 2:4. doi: 10.1186/s40694-015-0015-1 28955455 PMC5611662

[ref73] Meng G. Wang X. Liu M. Wang F. Liu Q. Dong C. (2022). Efficient CRISPR/Cas9 system based on autonomously replicating plasmid with an ama1 sequence and precisely targeted gene deletion in the edible fungus, cordyceps militaris. Microb. Biotechnol. 15, 2594–2606. doi: 10.1111/1751-7915.14107 35829671 PMC9518986

[ref74] Miao J. Li X. Lin D. Liu X. Tyler B. M. (2018). Oxysterol-binding protein-related protein 2 is not essential for Phytophthora sojae based on CRISPR/Cas9 deletions. Environ. Microbiol. Rep. 10, 293–298. doi: 10.1111/1758-2229.12638, PMID: 29521469

[ref75] Miller J. C. Tan S. Qiao G. Barlow K. A. Wang J. Xia D. F.. (2011). A tale nuclease architecture for efficient genome editing. Nat. Biotechnol. 29, 143–148. doi: 10.1038/nbt.1755, PMID: 21179091

[ref76] Miller S. M. Wang T. Randolph P. B. Arbab M. Shen M. W. Huang T. P.. (2020). Continuous evolution of SpCas9 variants compatible with non-G PAMs. Nat. Biotechnol. 38, 471–481. doi: 10.1038/s41587-020-0412-8, PMID: 32042170 PMC7145744

[ref77] Min K. Ichikawa Y. Woolford C. A. Mitchell A. P. (2016). *Candida albicans* gene deletion with a transient CRISPR-Cas9 system. Msphere. 1:e00130-16. doi: 10.1128/mSphere.00130-16, PMID: 27340698 PMC4911798

[ref78] Ming M. Ren Q. Pan C. He Y. Zhang Y. Liu S.. (2020). CRISPR-Cas12b enables efficient plant genome engineering. Nat. Plants. 6, 202–208. doi: 10.1038/s41477-020-0614-6, PMID: 32170285

[ref79] Moon S. An J. Y. Choi Y. J. Oh Y. L. Ro H. S. Ryu H. (2021). Construction of a CRISPR/Cas9-mediated genome editing system in Lentinula edodes. Mycobiology. 49, 599–603. doi: 10.1080/12298093.2021.2006401, PMID: 35035251 PMC8725921

[ref80] Nagy G. Vaz A. G. Szebenyi C. Tako M. Toth E. J. Csernetics A.. (2019). CRISPR-Cas9-mediated disruption of the HMG-CoA reductase genes of Mucor circinelloides and subcellular localization of the encoded enzymes. Fungal Genet. Biol. 129, 30–39. doi: 10.1016/j.fgb.2019.04.008, PMID: 30991115

[ref81] Nielsen M. L. Isbrandt T. Rasmussen K. B. Thrane U. Hoof J. B. Larsen T. O.. (2017). Genes linked to production of secondary metabolites in Talaromyces atroroseus revealed using CRISPR-Cas9. PLoS One 12:e169712. doi: 10.1371/journal.pone.0169712, PMID: 28056079 PMC5215926

[ref82] Nishida K. Arazoe T. Yachie N. Banno S. Kakimoto M. Tabata M.. (2016). Targeted nucleotide editing using hybrid prokaryotic and vertebrate adaptive immune systems. Science 353:aaf8729. doi: 10.1126/science.aaf8729, PMID: 27492474

[ref83] Nishimasu H. Shi X. Ishiguro S. Gao L. Hirano S. Okazaki S.. (2018). Engineered CRISPR-Cas9 nuclease with expanded targeting space. Science 361, 1259–1262. doi: 10.1126/science.aas9129, PMID: 30166441 PMC6368452

[ref84] Nodvig C. S. Hoof J. B. Kogle M. E. Jarczynska Z. D. Lehmbeck J. Klitgaard D. K.. (2018). Efficient oligo nucleotide mediated CRISPR-Cas9 gene editing in aspergilli. Fungal Genet. Biol. 115, 78–89. doi: 10.1016/j.fgb.2018.01.004, PMID: 29325827

[ref85] Nodvig C. S. Nielsen J. B. Kogle M. E. Mortensen U. H. (2015). A CRISPR-Cas9 system for genetic engineering of filamentous fungi. PLoS One 10:e133085. doi: 10.1371/journal.pone.0133085, PMID: 26177455 PMC4503723

[ref86] Pausch P. Al-Shayeb B. Bisom-Rapp E. Tsuchida C. A. Li Z. Cress B. F.. (2020). CRISPR-Casphi from huge phages is a hypercompact genome editor. Science 369, 333–337. doi: 10.1126/science.abb1400, PMID: 32675376 PMC8207990

[ref87] Peng R. Wang Y. Feng W. W. Yue X. J. Chen J. H. Hu X. Z.. (2018). CRISPR/dCas9-mediated transcriptional improvement of the biosynthetic gene cluster for the epothilone production in *Myxococcus xanthus*. Microb. Cell Factories 17:15. doi: 10.1186/s12934-018-0867-1 PMC5787926 29378572

[ref88] Phizicky E. M. Hopper A. K. (2010). tRNA biology charges to the front. Genes Dev. 24, 1832–1860. doi: 10.1101/gad.1956510, PMID: 20810645 PMC2932967

[ref89] Pohl C. Kiel J. A. Driessen A. J. Bovenberg R. A. Nygard Y. (2016). CRISPR/Cas9 based genome editing of Penicillium chrysogenum. ACS Synth. Biol. 5, 754–764. doi: 10.1021/acssynbio.6b00082, PMID: 27072635

[ref90] Prasad K. George A. Ravi N. S. Mohankumar K. M. (2021). CRISPR/Cas based gene editing: marking a new era in medical science. Mol. Biol. Rep. 48, 4879–4895. doi: 10.1007/s11033-021-06479-7, PMID: 34143395 PMC8212587

[ref91] Qi L. S. Larson M. H. Gilbert L. A. Doudna J. A. Weissman J. S. Arkin A. P.. (2013). Repurposing CRISPR as an RNA-guided platform for sequence-specific control of gene expression. Cell 152, 1173–1183. doi: 10.1016/j.cell.2013.02.022, PMID: 23452860 PMC3664290

[ref92] Qi L. S. Larson M. H. Gilbert L. A. Doudna J. A. Weissman J. S. Arkin A. P.. (2021). Repurposing CRISPR as an RNA-guided platform for sequence-specific control of gene expression. Cell 184:844. doi: 10.1016/j.cell.2021.01.019, PMID: 33545038

[ref93] Ran F. A. Cong L. Yan W. X. Scott D. A. Gootenberg J. S. Kriz A. J.. (2015). In vivo genome editing using *staphylococcus aureus* Cas9. Nature 520, 186–191. doi: 10.1038/nature14299, PMID: 25830891 PMC4393360

[ref94] Roux I. Woodcraft C. Hu J. Wolters R. Gilchrist C. Chooi Y. H. (2020). CRISPR-mediated activation of biosynthetic gene clusters for bioactive molecule discovery in filamentous fungi. ACS Synth. Biol. 9, 1843–1854. doi: 10.1021/acssynbio.0c00197, PMID: 32526136

[ref95] Sayari M. van der Nest M. A. Steenkamp E. T. Adegeye O. O. Marincowitz S. Wingfield B. D. (2019). Agrobacterium-mediated transformation of Ceratocystis albifundus. Microbiol. Res. 226, 55–64. doi: 10.1016/j.micres.2019.05.004, PMID: 31284945

[ref96] Sfeir A. Symington L. S. (2015). Microhomology-mediated end joining: a back-up survival mechanism or dedicated pathway? Trends Biochem.Sci. 40, 701–714. doi: 10.1016/j.tibs.2015.08.006 26439531 PMC4638128

[ref97] Shi T. Q. Gao J. Wang W. J. Wang K. F. Xu G. Q. Huang H.. (2019). CRISPR/Cas9-based genome editing in the filamentous fungus Fusarium fujikuroi and its application in strain engineering for gibberellic acid production. ACS Synth. Biol. 8, 445–454. doi: 10.1021/acssynbio.8b00478, PMID: 30616338

[ref98] Shi P. Murphy M. R. Aparicio A. O. Kesner J. S. Fang Z. Chen Z.. (2023). Collateral activity of the CRISPR/RfxCas13d system in human cells. Commun. Biol. 6:334. doi: 10.1038/s42003-023-04708-2 36977923 PMC10049998

[ref99] Song L. Ouedraogo J. P. Kolbusz M. Nguyen T. Tsang A. (2018). Efficient genome editing using tRNA promoter-driven CRISPR/Cas9 gRNA in Aspergillus niger. PLoS One 13:e202868. doi: 10.1371/journal.pone.0202868, PMID: 30142205 PMC6108506

[ref100] Song R. Zhai Q. Sun L. Huang E. Zhang Y. Zhu Y.. (2019). CRISPR/Cas9 genome editing technology in filamentous fungi: progress and perspective. Appl. Microbiol. Biotechnol. 103, 6919–6932. doi: 10.1007/s00253-019-10007-w, PMID: 31332488 PMC6690858

[ref101] Subhan M. Faryal R. Macreadie I. (2016). Exploitation of Aspergillus terreus for the production of natural statins. J. Fungi. 2:13. doi: 10.3390/jof2020013, PMID: 29376930 PMC5753075

[ref102] Sugano S. S. Suzuki H. Shimokita E. Chiba H. Noji S. Osakabe Y.. (2017). Genome editing in the mushroom-forming basidiomycete Coprinopsis cinerea, optimized by a high-throughput transformation system. Sci. Rep. 7:1260. doi: 10.1038/s41598-017-00883-5 28455526 PMC5430836

[ref103] Sullivan C. F. Parker B. L. Skinner M. (2022). A review of commercial metarhizium- and beauveria-based biopesticides for the biological control of ticks in the USA. Insects. 13:260. doi: 10.3390/insects13030260, PMID: 35323558 PMC8952794

[ref104] Sun A. Li C. P. Chen Z. Zhang S. Li D. Y. Yang Y.. (2023). The compact Caspi (Cas12l) 'bracelet' provides a unique structural platform for DNA manipulation. Cell Res. 33, 229–244. doi: 10.1038/s41422-022-00771-2, PMID: 36650285 PMC9977741

[ref105] Szczelkun M. D. Tikhomirova M. S. Sinkunas T. Gasiunas G. Karvelis T. Pschera P.. (2014). Direct observation of r-loop formation by single RNA-guided Cas9 and cascade effector complexes. Proc. Natl. Acad. Sci. USA 111, 9798–9803. doi: 10.1073/pnas.1402597111, PMID: 24912165 PMC4103346

[ref106] Tong H. Huang J. Xiao Q. He B. Dong X. Liu Y.. (2023). High-fidelity Cas13 variants for targeted RNA degradation with minimal collateral effects. Nat. Biotechnol. 41, 108–119. doi: 10.1038/s41587-022-01419-7, PMID: 35953673

[ref107] Tu J. L. Bai X. Y. Xu Y. L. Li N. Xu J. W. (2021). Targeted gene insertion and replacement in the basidiomycete Ganoderma lucidum by inactivation of nonhomologous end joining using CRISPR/Cas9. Appl. Environ. Microbiol. 87:e151021. doi: 10.1128/AEM.01510-21, PMID: 34524900 PMC8579997

[ref108] Vanegas K. G. Jarczynska Z. D. Strucko T. Mortensen U. H. (2019). Cpf1 enables fast and efficient genome editing in aspergilli. Fungal Biol Biotechnol. 6:6. doi: 10.1186/s40694-019-0069-6 31061713 PMC6492335

[ref109] Vanegas K. G. Rendsvig J. Jarczynska Z. D. Cortes M. van Esch A. P. Morera-Gomez M.. (2022). A Mad7 system for genetic engineering of filamentous fungi. J. Fungi. 9:16. doi: 10.3390/jof9010016 PMC9865164 36675838

[ref110] Vojta A. Dobrinic P. Tadic V. Bockor L. Korac P. Julg B.. (2016). Repurposing the CRISPR-Cas9 system for targeted DNA methylation. Nucleic Acids Res. 44, 5615–5628. doi: 10.1093/nar/gkw159, PMID: 26969735 PMC4937303

[ref111] Vyas V. K. Bushkin G. G. Bernstein D. A. Getz M. A. Sewastianik M. Barrasa M. I.. (2018). New CRISPR mutagenesis strategies reveal variation in repair mechanisms among fungi. Msphere. 3:e00154-18. doi: 10.1128/mSphere.00154-18, PMID: 29695624 PMC5917429

[ref112] Walton R. T. Christie K. A. Whittaker M. N. Kleinstiver B. P. (2020). Unconstrained genome targeting with near-PAMless engineered CRISPR-Cas9 variants. Science 368, 290–296. doi: 10.1126/science.aba8853, PMID: 32217751 PMC7297043

[ref113] Wang Q. Cobine P. A. Coleman J. J. (2018). Efficient genome editing in Fusarium oxysporum based on CRISPR/Cas9 ribonucleoprotein complexes. Fungal Genet. Biol. 117, 21–29. doi: 10.1016/j.fgb.2018.05.003, PMID: 29763675 PMC6480338

[ref114] Wang Q. Coleman J. J. (2019). Progress and challenges: development and implementation of CRISPR/Cas9 technology in filamentous fungi. Comp. Struct. Biotechnol. J. 17, 761–769. doi: 10.1016/j.csbj.2019.06.007, PMID: 31312414 PMC6607083

[ref115] Wang P. A. Xiao H. Zhong J. J. (2020). CRISPR-Cas9 assisted functional gene editing in the mushroom Ganoderma lucidum. Appl. Microbiol. Biotechnol. 104, 1661–1671. doi: 10.1007/s00253-019-10298-z, PMID: 31865439

[ref116] Wenderoth M. Pinecker C. Voss B. Fischer R. (2017). Establishment of CRISPR/Cas9 in *Alternaria alternata*. Fungal Genet. Biol. 101, 55–60. doi: 10.1016/j.fgb.2017.03.001, PMID: 28286319

[ref117] Wosten H. (2019). Filamentous fungi for the production of enzymes, chemicals and materials. Curr. Opin. Biotechnol. 59, 65–70. doi: 10.1016/j.copbio.2019.02.010, PMID: 30901669

[ref118] Wu C. Chen Y. Qiu Y. Niu X. Zhu N. Chen J.. (2020). A simple approach to mediate genome editing in the filamentous fungus Trichoderma reesei by CRISPR/Cas9-coupled in vivo gRNA transcription. Biotechnol. Lett. 42, 1203–1210. doi: 10.1007/s10529-020-02887-0, PMID: 32300998

[ref119] Wu T. Liu C. Zou S. Lyu R. Yang B. Yan H.. (2023). An engineered hypercompact CRISPR-Cas12f system with boosted gene-editing activity. Nat. Chem. Biol. 10:1038. doi: 10.1038/s41589-023-01380-9 PMC10625714 37400536

[ref120] Wu Z. Zhang Y. Yu H. Pan D. Wang Y. Wang Y.. (2021). Programmed genome editing by a miniature CRISPR-Cas12f nuclease. Nat. Chem. Biol. 17, 1132–1138. doi: 10.1038/s41589-021-00868-6, PMID: 34475565

[ref121] Xiao B. Yin S. Hu Y. Sun M. Wei J. Huang Z.. (2019). Epigenetic editing by CRISPR/dCas9 in plasmodium falciparum. Proc. Natl. Acad. Sci. USA 116, 255–260. doi: 10.1073/pnas.1813542116, PMID: 30584102 PMC6320497

[ref122] Xin C. Yin J. Yuan S. Ou L. Liu M. Zhang W.. (2022). Comprehensive assessment of miniature CRISPR-Cas12f nucleases for gene disruption. Nat. Commun. 13:5623. doi: 10.1038/s41467-022-33346-1 36153319 PMC9509373

[ref123] Xu C. L. Ruan M. Mahajan V. B. Tsang S. H. (2019). Viral delivery systems for CRISPR. Viruses-Basel. 11:28. doi: 10.3390/v11010028, PMID: 30621179 PMC6356701

[ref124] Xu C. Zhou Y. Xiao Q. He B. Geng G. Wang Z.. (2021). Programmable RNA editing with compact CRISPR-Cas13 systems from uncultivated microbes. Nat. Methods 18, 499–506. doi: 10.1038/s41592-021-01124-4, PMID: 33941935

[ref125] Yan W. X. Chong S. Zhang H. Makarova K. S. Koonin E. V. Cheng D. R.. (2018). Cas13d is a compact RNA-targeting type VI CRISPR effector positively modulated by a WYL-domain-containing accessory protein. Mol. Cell 70, 327–339. doi: 10.1016/j.molcel.2018.02.028, PMID: 29551514 PMC5935466

[ref126] Yan W. X. Hunnewell P. Alfonse L. E. Carte J. M. Keston-Smith E. Sothiselvam S.. (2019). Functionally diverse type V CRISPR-Cas systems. Science 363, 88–91. doi: 10.1126/science.aav7271, PMID: 30523077 PMC11258546

[ref127] Youssar L. Wernet V. Hensel N. Yu X. Hildebrand H. G. Schreckenberger B.. (2019). Intercellular communication is required for trap formation in the nematode-trapping fungus Duddingtonia flagrans. PLoS Genet. 15:e1008029. doi: 10.1371/journal.pgen.1008029 30917129 PMC6453484

[ref128] Yu X. Ji S. L. He Y. L. Ren M. F. Xu J. W. (2014). Development of an expression plasmid and its use in genetic manipulation of Lingzhi or Reishi medicinal mushroom, Ganoderma lucidum (higher basidiomycetes). Int. J. Med. Mushrooms. 16, 161–168. doi: 10.1615/IntJMedMushr.v16.i2.60, PMID: 24941037

[ref129] Zetsche B. Gootenberg J. S. Abudayyeh O. O. Slaymaker I. M. Makarova K. S. Essletzbichler P.. (2015). Cpf1 is a single RNA-guided endonuclease of a class 2 CRISPR-Cas system. Cell 163, 759–771. doi: 10.1016/j.cell.2015.09.038, PMID: 26422227 PMC4638220

[ref130] Zhang C. Li N. Rao L. Li J. Liu Q. Tian C. (2022). Development of an efficient C-to-T base-editing system and its application to cellulase transcription factor precise engineering in thermophilic fungus Myceliophthora thermophila. Microbiol. Spectr. 10:e232121. doi: 10.1128/spectrum.02321-21, PMID: 35608343 PMC9241923

[ref131] Zhang C. Meng X. Wei X. Lu L. (2016). Highly efficient CRISPR mutagenesis by microhomology-mediated end joining in *Aspergillus fumigatus*. Fungal Genet. Biol. 86, 47–57. doi: 10.1016/j.fgb.2015.12.007, PMID: 26701308

[ref132] Zhang Y. Rajan R. Seifert H. S. Mondragon A. Sontheimer E. J. (2015). DNase H activity of *Neisseria meningitidis* Cas9. Mol. Cell 60, 242–255. doi: 10.1016/j.molcel.2015.09.020, PMID: 26474066 PMC4609032

[ref133] Zhang L. Zheng X. Cairns T. C. Zhang Z. Wang D. Zheng P.. (2020). Disruption or reduced expression of the orotidine-5′-decarboxylase gene pyrG increases citric acid production: a new discovery during recyclable genome editing in Aspergillus niger. Microb. Cell Factories 19:76. doi: 10.1186/s12934-020-01334-z PMC7092557 32209089

[ref134] Zheng X. Zheng P. Zhang K. Cairns T. C. Meyer V. Sun J.. (2019). 5s rRNA promoter for guide RNA expression enabled highly efficient CRISPR/Cas9 genome editing in Aspergillus niger. ACS Synth. Biol. 8, 1568–1574. doi: 10.1021/acssynbio.7b00456, PMID: 29687998

[ref135] Zou G. Xiao M. Chai S. Zhu Z. Wang Y. Zhou Z. (2021). Efficient genome editing in filamentous fungi via an improved CRISPR-Cas9 ribonucleoprotein method facilitated by chemical reagents. Microb. Biotechnol. 14, 2343–2355. doi: 10.1111/1751-7915.13652, PMID: 32841542 PMC8601184

